# A regulatory loop of JAK/STAT signalling and its downstream targets represses cell fate conversion and maintains male germline stem cell niche homeostasis

**DOI:** 10.1111/cpr.13648

**Published:** 2024-07-10

**Authors:** Ruiyan Kong, Hang Zhao, Juan Li, Yankun Ma, Ningfang Li, Lin Shi, Zhouhua Li

**Affiliations:** ^1^ Laboratory of Stem Cell Biology, College of Life Sciences Capital Normal University Beijing China

## Abstract

A specialised microenvironment, termed niche, provides extrinsic signals for the maintenance of residential stem cells. However, how residential stem cells maintain niche homeostasis and whether stromal niche cells could convert their fate into stem cells to replenish lost stem cells upon systemic stem cell loss remain largely unknown. Here, through systemic identification of JAK/STAT downstream targets in adult *Drosophila* testis, we show that Escargot (Esg), a member of the Snail family of transcriptional factors, is a putative JAK/STAT downstream target. *esg* is intrinsically required in cyst stem cells (CySCs) but not in germline stem cells (GSCs). *esg* depletion in CySCs results in CySC loss due to differentiation and non‐cell autonomous GSC loss. Interestingly, hub cells are gradually lost by delaminating from the hub and converting into CySCs in *esg*‐*defective* testes. Mechanistically, *esg* directly represses the expression of *socs36E*, the well‐known downstream target and negative regulator of JAK/STAT signalling. Finally, further depletion of *socs36E* completely rescues the defects observed in *esg*‐*defective* testes. Collectively, JAK/STAT target Esg suppresses SOCS36E to maintain CySC fate and repress niche cell conversion. Thus, our work uncovers a regulatory loop between JAK/STAT signalling and its downstream targets in controlling testicular niche homeostasis under physiological conditions.

## Graphical abstract

Esg suppresses the expression of SOCS36E, the downstream target and negative regulator of JAK/STAT signalling. Loss of *esg* leads to systemic CySC loss. Once the hub senses the systemic loss of CySCs, some hub cells are delaminated from the hub and converted into functional CySCs to replace the lost CySCs.

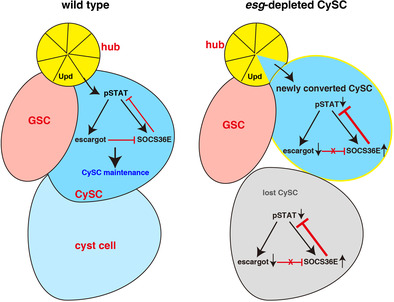

## 1 INTRODUCTION

The perfect balance between self‐renewal and differentiation in adult stem cells is necessary to maintain tissue homeostasis. The capacity of adult stem cells to undergo proper self‐renewal and differentiation requires suitable intrinsic cellular determinants and extrinsic signals derived from the local specialized microenvironment, also known as the ‘niche’.^1^, ^2^, ^3^, ^4^, ^5^, ^6^ The stem cell niche plays critical roles in regulating stem cell maintenance, self‐renewal, and differentiation.^7^, ^8^, ^9^, ^10^, ^11^, ^12^, ^13^ The physical contact between stem cells and stromal niche cells or the extracellular matrix keeps stem cells within the niche and close to extrinsic signals that allow stem cells to constantly self‐renew and produce differentiated progeny. Accordingly, many niche cells (like the hub mentioned below) are thought to be post‐mitotic and quiescent under physiological conditions, while under some non‐physiological conditions, niche cells could be lost, expanded, or converted into stem cells.^14^, ^15^, ^16^, ^17^, ^18^, ^19^, ^20^, ^21^, ^22^ However, little is known about how niche homeostasis is maintained, especially the factors regulating stromal niche cells to convert their fate into stem cells. Elucidating the underlying molecular mechanisms will lead to the development of new regenerative and anti‐tumour/aging therapies.

The adult *Drosophila* testis provides an ideal model system for characterising the maintenance of stem cells/niche and the relationship between stem cell behaviour and the niche due to its powerful molecular genetic tools and the conservation of various signalling pathways.^5^, ^23^, ^24^ At the tip of the *Drosophila* testis, a group of somatic cells, termed hub, are physically in contact with two stem cell populations: germline stem cells (GSCs) and somatic cyst stem cells (CySCs).^23^, ^25^, ^26^, ^27^ The hub cells serve as the niche for both GSCs and CySCs by secreting factors to regulate their self‐renewal and differentiation.^28^, ^29^, ^30^ GSC divides asymmetrically to produce a new GSC and a daughter cell called gonialblast(GB) which will be finally differentiated into mature sperm.^31^, ^32^ Meanwhile, CySCs self‐renew and generate cyst cells to encapsulate the GB and begin to differentiate without proliferation in concert with the germ cells.^22^, ^33^, ^34^, ^35^, ^36^ Using single‐nucleus RNA‐seq (snRNA‐seq) and new single‐cell RNA‐seq (scRNA‐seq) data, an extensive characterisation of cell types found within the *Drosophila* testis was recently provided.^37^ Hub cells and CySCs are derived from a common group of ancestor cells and specified early during embryogenesis.^38^, ^39^ Hub cells are regarded as quiescent in adult testes, however, in some circumstances, like upon systemic CySC ablation, depleting the cell cycle inhibitor and tumour suppressor retinoblastoma (RB), or ectopic expression of Cyclin D‐Cdk4 in the hub, hub cells will quit the quiescent state and convert their fate into functional CySCs.^14^, ^16^, ^20^, ^21^ Meanwhile, the cell‐cycle‐responsive Dp/E2f1 transcription factor is required in CySCs to non‐autonomously maintain hub cell quiescence by inhibiting local Activin receptor signalling in hub cells through production of the Activin antagonist Follistatin (Fs).^40^ However, it remains not fully understood how new CySCs are regenerated upon gradual and systemic CySC loss.

Several conserved signalling pathways, including the JAK/STAT pathway, regulate GSC and/or CySC maintenance in the testis.^28^, ^30^, ^41^, ^42^, ^43^, ^44^, ^45^, ^46^, ^47^, ^48^, ^49^, ^50^, ^51^, ^52^, ^53^ The hub secretes the ligand Upd (Unpaired) which activates JAK/STAT signalling in the adjacent CySCs to regulate their maintenance.^28^, ^30^, ^36^ Activation of JAK/STAT signalling in CySCs is also important for GSC self‐renewal in a non‐cell autonomous manner, thereby CySCs constitute a major niche component for GSCs.^29^, ^54^, ^55^ The JAK/STAT downstream targets, such as *Zinc‐finger homeodomain protein 1* (*Zfh1*) and *chronologically inappropriate morphogenesis* (*Chinmo*), are intrinsically required for CySC maintenance and non‐cell autonomously for GSC maintenance.^29^, ^54^ Suppressors of cytokine signalling (SOCS) are the highly conserved transcriptional targets of JAK/STAT signalling and negatively regulate JAK/STAT signalling via distinct mechanisms.^56^, ^57^, ^58^ SOCS36E is expressed in the hub and CySCs and is reported to inhibit ERK activity in CySCs to prevent them from outcompeting neighbouring GSCs.^36^, ^59^, ^60^, ^61^, ^62^ However, whether additional JAK/STAT downstream targets are intrinsically required for CySC self‐renewal and niche homeostasis remains to be studied.

**FIGURE 1:**
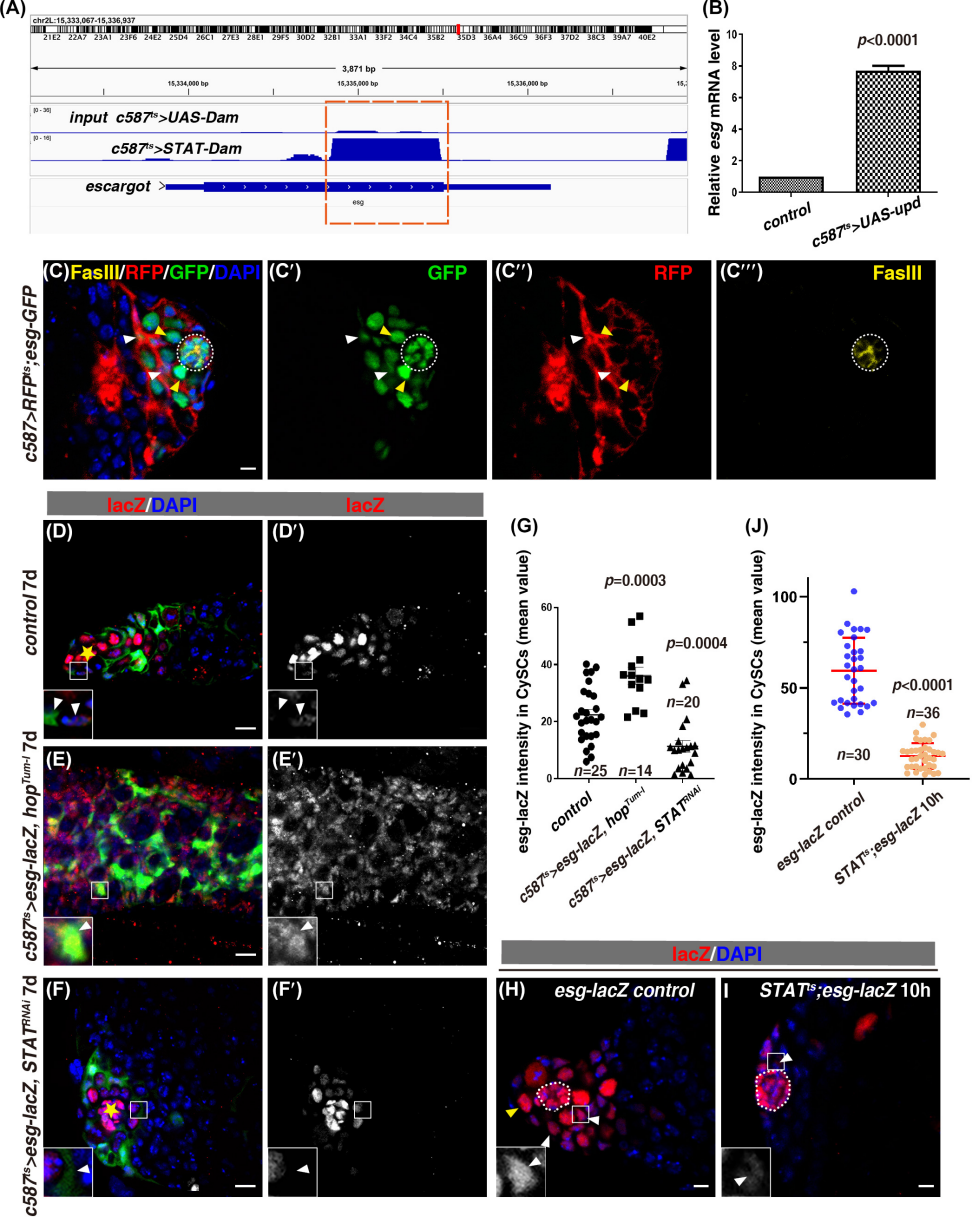
*esg* is a putative JAK/STAT downstream target in *Drosophila* testis. (A) Dam‐ID analysis for STAT (Stat92E)‐Dam and control reveals binding peaks of STAT at the *esg* region (orange dashed box). (B) qRT–PCR quantification of *esg* mRNA levels in testes with indicated genotypes. Mean ± SD is shown. Two‐tailed Student's *t‐*test was used. The *p*‐value is indicated in the graph (the same as follows). *n* = 3. (C) Esg (in green by *esg‐GFP*) is expressed in the hub (white dotted cycle, by FasIII in yellow), early germline cells (yellow arrowheads), and early cyst cells (white arrowheads). The somatic cyst cells are marked by *c587 > RFP* ^*ts*^ (red). Separated channels are shown. (D)–(F) *esg‐lacZ* (red) in testes from *c587* ^*ts*^ *>w* ^*v20*^ (control), *c587* ^*ts*^ *>hop* ^*Tum‐l*^, and *c587* ^*ts*^ *>STAT* ^*RNAi*^ at 29°C for 7 days. *esg‐lacZ* channel is shown separately in black and white. Enlarged region is shown in the white box. The hub is indicated by yellow asterisk. UAS‐GFP is expressed by *c587* ^*ts*^ to label somatic cyst cell lineage (the same as follows unless otherwise specified). (G) Quantification of *esg*‐lacZ fluorescence intensity in CySCs in testes with indicated genotypes. Mean ± SD is shown. Ordinary one‐way ANOVA test was used. The number (*n*) is indicated in the graph (the same as follows). (H), (I) *esg‐lacZ* in control and *STAT* ^*ts*^ testes at 29°C for 10 h. White dotted cycle marks the hub, GSCs, and CySCs are indicated by yellow and white arrowheads respectively. Boxed region is shown in high magnification. (J) Quantification of *esg*‐lacZ intensity in CySCs from testes with indicated genotypes. Mean ± SD is shown. Two‐tailed student's *t‐*test was used. The nucleus is stained by DAPI in blue in all confocal images. Scale bars: 5 μm and 10 μm (D)–(F).

To systematically identify additional downstream targets of JAK/STAT signalling in adult testis, we utilised adenine methylase identification (Dam‐ID) technology.^51^, ^63^, ^64^, ^65^ *escargot* (*esg*) was identified as a potential JAK/STAT downstream target in this assay (Figure 1A, B). Esg is a member of the Snail family of transcription factors,^66^, ^67^ it plays important roles in epithelial‐mesenchymal transition, stem cell fate determination, cell movement, and survival in mammals.^67^, ^68^, ^69^, ^70^, ^71^, ^72^, ^73^, ^74^ In *Drosophila*, Esg is essential for various aspects of developmental processes, such as tracheal tube formation and the maintenance of intestinal stem cells (ISCs).^66^, ^75^, ^76^, ^77^, ^78^, ^79^ In adult *Drosophila* testis, Esg is expressed in the hub, early germline, and somatic cyst cells (Figure 1C), *esg‐defective* hub cells convert (transdifferentiate) into CySCs, leading to hub depletion.^16^ Meanwhile, we noted that during the course of this study, it was reported that *esg* is autonomously required for CySC maintenance, in which *esg* is claimed to function through the Insulin signalling.^16^, ^80^ However, how *esg* is regulated and how it controls niche homeostasis await further exploration.

In this study, we show that *esg* is a putative downstream target of JAK/STAT signalling. *esg* is intrinsically required in CySCs for the maintenance of CySCs and GSCs. Interestingly, hub cells respond to systemic CySC loss by converting their fate into CySC to maintain niche homeostasis. Mechanistically, we show that *socs36E* is a downstream target of Esg, and its repression by Esg is responsible for the defects observed in testes with *esg‐defective* CySCs. Thus, our data reveal a regulatory loop between JAK/STAT signalling and its downstream targets in niche cell conversion and niche homeostasis maintenance.

## 2 RESULTS

### 2.1 *esg* is a putative JAK/STAT downstream target in adult *Drosophila* testis

JAK/STAT signalling is important for the self‐renewal and differentiation of CySCs.^28^, ^30^ To systemically search for new downstream targets of JAK/STAT signalling in cyst cells, we utilised the Dam‐ID technique to seek genome‐wide binding sites of Stat92E (STAT) in vivo.^51^, ^63^, ^64^, ^65^ A STAT‐DNA methylase fusion protein (STAT‐Dam) will enrich DNA methylation at genomic regions where the activated STAT‐Dam binds to. A STAT‐Dam was expressed in the cyst cells by the *c587* ^*ts*^ (*c587Gal4, UAS‐GFP, tubGal80* ^*ts*^) driver, and enriched STAT‐binding regions were defined by comparing STAT‐Dam methylation profiles to a Dam‐alone control. The STAT‐Dam‐ID assay was proved to be successful as known JAK/STAT targets and a cohort of novel putative targets were identified.^51^ Dam‐ID sequencing data analysis revealed binding peaks of STAT in *escargot* (*esg*) region, indicating that *esg* is a potential downstream target of JAK/STAT signalling (Figure 1A). To confirm these results, we further examined the expression levels of *esg* upon activation of JAK/STAT signalling by quantitative real‐time reverse transcriptase‐PCR (qRT‐PCR). The qRT‐PCR results showed that the mRNA levels of *esg* were significantly upregulated upon *upd* overexpression in cyst cells (Figure 1B). Esg is a member of the Snail family of transcriptional repressors and is expressed in hub cells, early germline, and cyst cells at the tip of the adult testis (Figure 1C).^16^, ^67^, ^80^, ^81^, ^82^ In order to test whether *esg* is positively regulated by JAK/STAT signalling, the expression levels of *esg* in CySCs were further examined by comparing the mean fluorescence intensity of *esg‐lacZ* in CySCs. The data showed that the expression levels of *esg‐lacZ* in somatic cyst cells were also significantly elevated upon activation of JAK/STAT signalling by *hop* ^*Tum‐l*^ overexpression, a constitutively active form of JAK^83^; on the contrary, the levels of *esg‐lacZ* in CySCs were diminished upon STAT depletion (Figure 1D–G). Moreover, the levels of *esg‐lacZ* in CySCs were dramatically reduced in *STAT* ^*ts*^ mutant compared with those in the control (Figure 1H–J). Altogether, these data show that *esg* is a putative downstream target of JAK/STAT signalling in CySCs.

### 2.2 *esg* is necessary to maintain CySC fate

**FIGURE 2:**
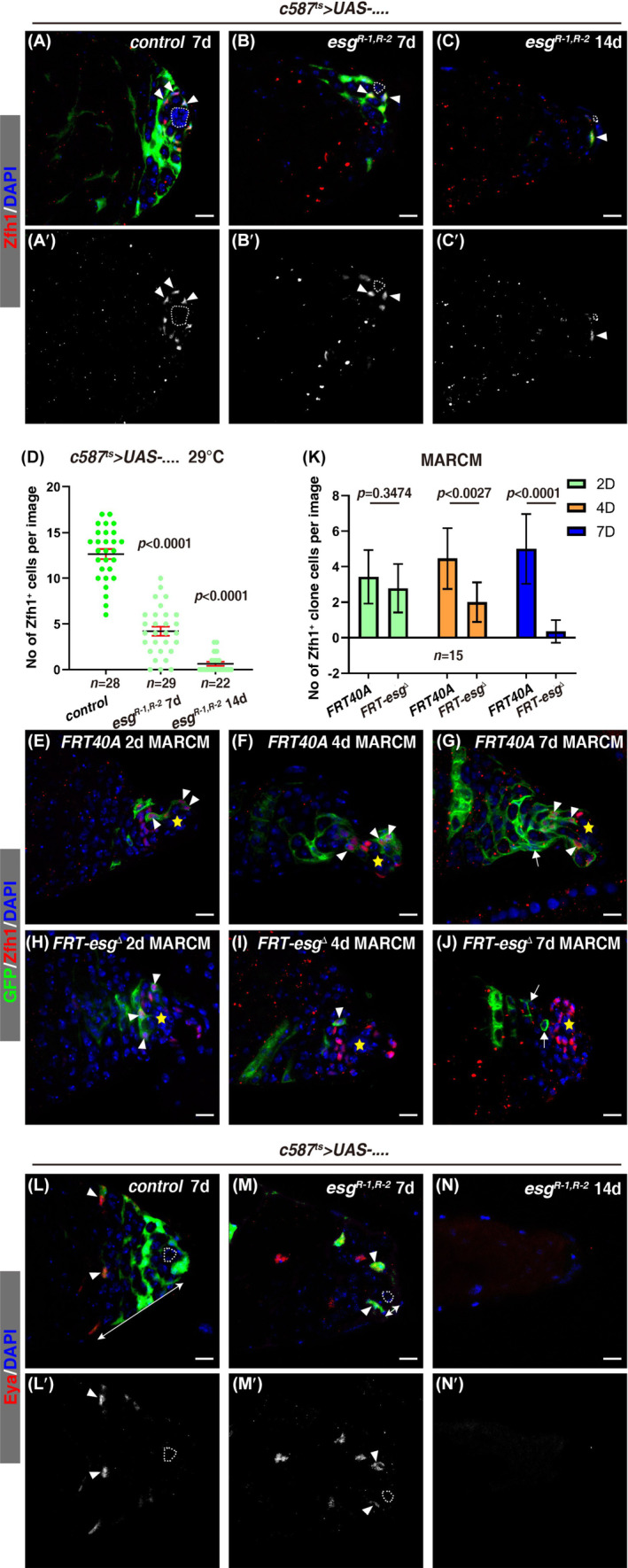
*esg* is required for CySC maintenance. (A)–(C) Zfh1 staining (red, white arrowheads) in control and *c587* ^*ts*^ *>esg* ^*RNAi*^ testes at 29°C for 7 days or 14 days. Zfh1 channel is shown separately in black and white. (D) Quantification of the number of Zfh1^+^ cells/image in testes with indicated genotypes. Mean ± SD is shown. Ordinary one‐way ANOVA test was used. (E)–(J) Zfh1 staining (red, white arrowheads) in control and *esg* ^*Δ*^ CySC MARCM clones at 2, 4, and 7 days after clone induction (ACI). The differentiated cyst cells are indicated by white arrows. (*K*) Quantification of the number of Zfh1^+^ cells/clone in testes with indicated genotypes at pointed time points. Mean ± SD is shown. Mixed two‐way ANOVA test was used. (L)–(N) Eya staining (red, white arrowheads) in control and *esg* ^*RNAi*^ testes. The white lines with double arrowheads indicate the distance between Eya^+^ mature cyst cells and the hub. Eya channel is shown separately in black and white. In all confocal images, white dotted cycles or yellow asterisks mark the hub, and DAPI is stained for the nucleus in blue. Scale bars: 10 μm.

To examine the function of *esg* in somatic cyst cells in adult testis in detail, we depleted *esg* via different effective RNAi constructs against *esg* using *c587* ^*ts*^ driver which is expressed in somatic cyst cell lineage (SI Appendix, Figure S1). We stained the testes with anti‐Zfh1 antibody, which labels CySCs and early cyst cells. Compared with control, depletion of *esg* in somatic cyst cells using individual RNAi construct resulted in gradual reduction of CySCs (Zfh1^+^) and early cyst cells, and eventual loss of all *c587>GFP* ^+^ cyst cells, while depleting *esg* by simultaneous induction of two RNAi constructs caused more rapid loss of CySCs (Figure 2A–D and SI Appendix, Figure S2).^29^, ^50^ These data suggest that *esg* may be required for CySC maintenance or viability. To exclude the possibility of an off‐target effect, we performed rescue experiments. Overexpression of *esg* resulted in great accumulation of TJ^+^ CySC‐like cells and the absence of Eya^+^ differentiated cyst cells (SI Appendix, Figure S3A–F). The number of PH3^+^ somatic cyst cells per testis also was increased (SI Appendix, Figure S3G–I). These data suggest that overexpression of *esg* in cyst cells promotes CySC proliferation. Furthermore, overexpression of *esg* completely rescued the defects caused by *esg* depletion, indicating that the CySC‐defective phenotype is indeed caused by loss of *esg* (SI Appendix, Figure S3J–N). To further confirm the results obtained by *esg* ^*RNAi*^ lines, we generated *esg* null mutant by Δ2‐3 transposase (*esg* ^*Δ*^, SI Appendix, Figure S4A) and performed MARCM (mosaic analysis with a repressible cell marker) clonal analysis.^84^ Compared with control CySC MARCM clones, *esg* ^*Δ*^ mutant CySC MARCM clones were quickly lost by 7 days after clone induction (ACI) (SI Appendix, Figure S4B, C). We conducted time course MARCM analysis to further examine the loss of *esg* mutant CySC MARCM clones. The control CySC MARCM clones contained at least one Zfh1^+^ CySC at 2 days, 4 days, or 7 days ACI (Figure 2E–G). Whilst the average number of Zfh1^+^ *esg* ^*Δ*^ CySC clones was similar to that of control CySC clones 2 days ACI, the number of Zfh1^+^ *esg* ^*Δ*^ CySC clones was decreased at 4 days ACI, eventually about 80% *esg* ^*Δ*^ CySC MARCM clones were devoid of Zfh1^+^ cells, indicative of CySC loss (Figure 2H–K). These data suggest that the *esg* ^*Δ*^ mutant CySC MARCM clones are gradually lost. We further examined the testes with an antibody against the large Maf factor Traffic Jam (TJ) which is highly expressed in CySCs and early cyst cells.^85^ Supporting this notion, the number of TJ^+^ cyst cells was also diminished in *c587* ^*ts*^ *>esg* ^*RNAi*^ testes (SI Appendix, Figure S5A–D). Collectively, these data indicate that *esg‐defective* CySCs cannot maintain their CySC fate, which is consistent with previous report.^16^, ^80^

The great reduction of CySCs observed in *c587* ^*ts*^ *>esg* ^*RNAi*^ testes may be due to precocious differentiation and/or cell death. To discriminate these possibilities, we first examined the testes with antibodies against the mature cyst cell marker, Eyes absent (Eya).^86^ Compared with the control testes in which mature Eya^+^ cyst cells are far away from the hub, however, Eya^+^ cyst cells in *c587* ^*ts*^ *>esg* ^*RNAi*^ testes finally occupied the niche region, indicating that *esg‐defective* CySCs cannot be maintained and differentiated into cyst cells (Figure 2L–N and SI Appendix, Figure S5E–M). We further examined cell death in the *esg*‐defective cyst cells using ‘GC3Ai’, a fluorescent sensor for caspase activity.^87^ Compared to the GFP^+^ signals found in apoptotic cells by overexpressing *reaper* (*rpr*), the apoptosis inducer, cell death in cyst cells of *c587* ^*ts*^ *>esg* ^*RNAi*^ testes was largely unaffected (SI Appendix, Figure S5N–P).^87^ Collectively, these data indicate that *esg* is intrinsically required for CySC maintenance under physiological conditions, which is consistent with previous report.^16^, ^80^

### 2.3 Loss of *esg* in CySCs non‐cell autonomously affects GSC and hub maintenance

**FIGURE 3:**
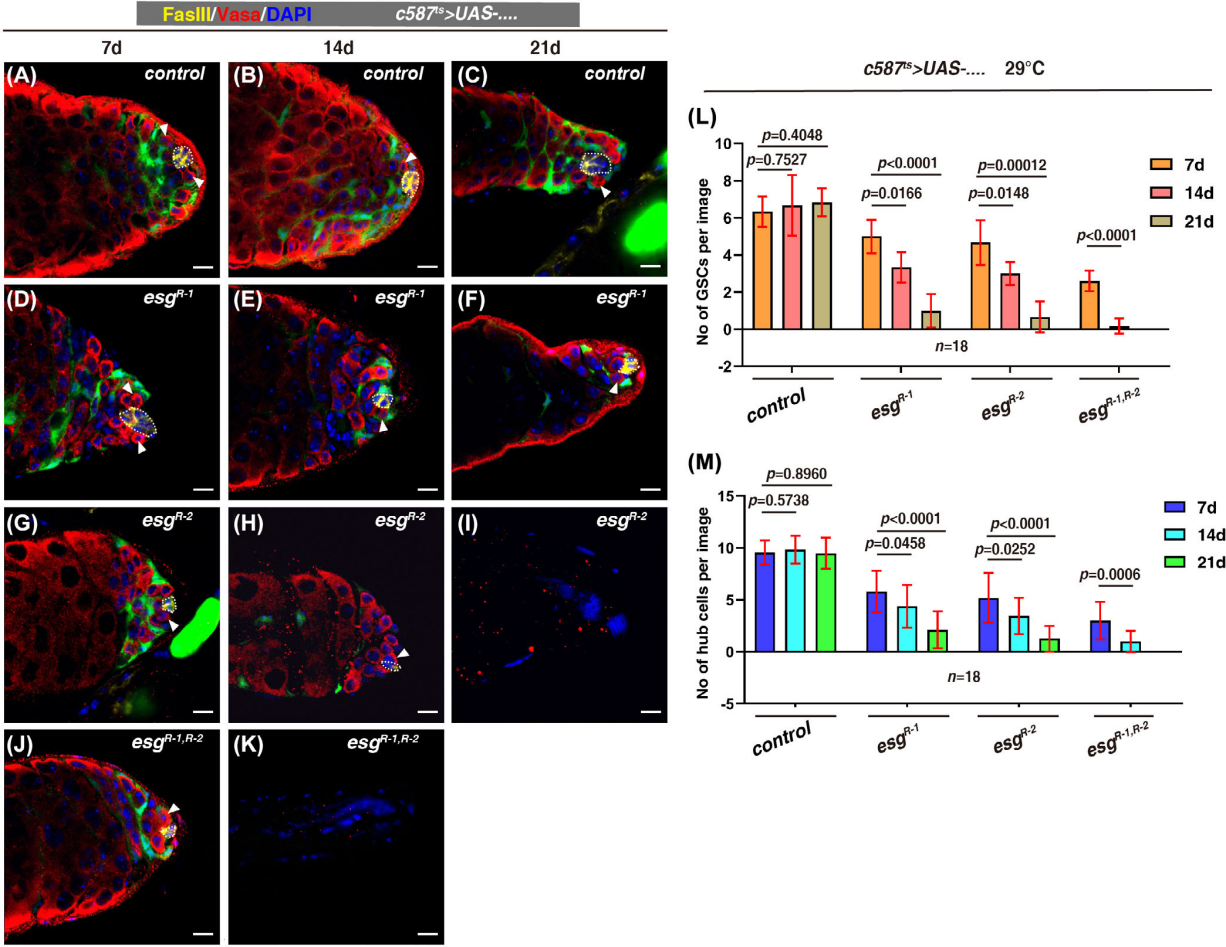
Loss of *esg* in CySCs non‐cell autonomously affects GSC and hub maintenance. (A)–(K) Vasa (red) and FasIII (yellow) staining in control and *c587* ^*ts*^ *>esg* ^*RNAi*^ testes at 29°C for 7, 14, and 21 days, respectively. GSC is indicated by white arrowhead, the hub is indicated by white dotted cycle, and DAPI labels the nucleus in blue. Scale bars: 10 μm. (L) Quantification of the number of GSCs/image in testes with indicated genotypes. Mean ± SD is shown. Multiple Student's *t*‐test was used. (M) Quantification of the number of hub cells/image in testes with indicated genotypes. Mean ± SD is shown. Multiple Student's *t‐*tes*t* was used.

As CySCs are part of the GSC niche and required for GSC self‐renewal and differentiation in a non‐cell autonomous manner,^10^, ^27^, ^29^, ^34^, ^54^, ^55^ we then examined whether GSC maintenance was non‐cell autonomously affected in *c587* ^*ts*^>*esg* ^*RNAi*^ testes. We found that the number of GSCs was gradually decreased and germline cells were totally lost in *c587* ^*ts*^>*esg* ^*RNAi*^ testes eventually (Figure 3A–L). Consistently, the number of cells with high levels of STAT surrounding the hub was significantly reduced in *c587* ^*ts*^>*esg* ^*RNAi*^ testes (SI Appendix, Figure S6). These data show that *esg* is non‐cell autonomously required for GSC maintenance. Interestingly, we found that hub cells were also gradually lost and niche homeostasis was totally disrupted eventually in *c587* ^*ts*^>*esg* ^*RNAi*^ testes, suggesting that there is a communication between the hub and CySCs to maintain niche homeostasis upon systemic CySC/GSC loss (Figure 3A–K, M and SI Appendix, Figure S5, S6). We also examined whether *esg* is intrinsically required for GSC behaviour as it is also highly expressed in GSCs. However, no obvious defects were observed when *esg* was depleted in germline cells, indicating that *esg* is likely dispensable for GSC maintenance (SI Appendix, Figure S7). Altogether, these data demonstrate that *esg* functions in somatic cyst cells and is essential for maintaining niche homeostasis.

### 2.4 Hub cells are converted into CySCs upon loss of *esg* in CySCs

**FIGURE 4:**
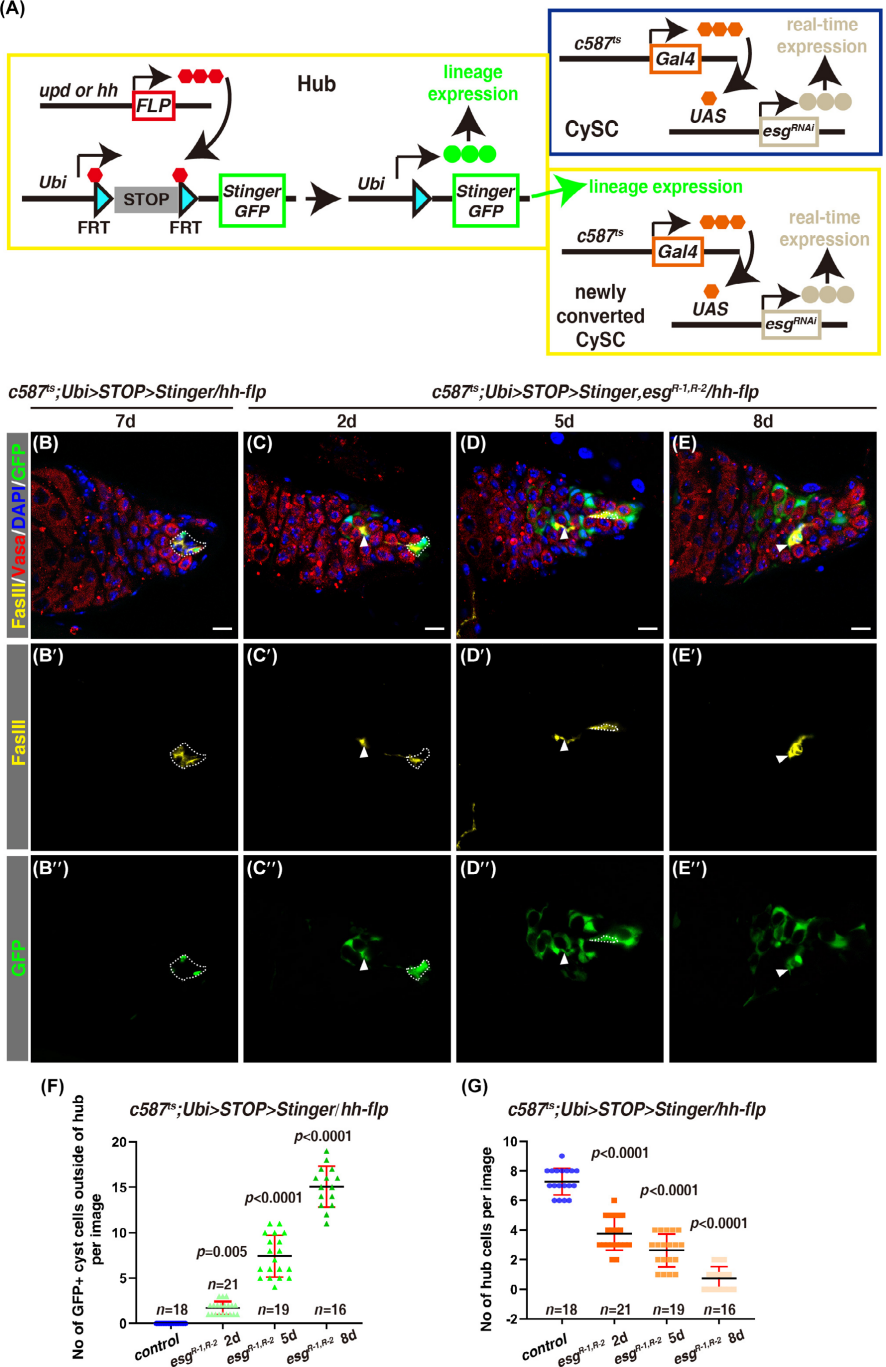
Hub cells convert into functional CySCs upon loss of *esg* in CySCs. (A) The schematic diagram of G‐TRACE method developed to trace the hub lineage. FLP is specifically expressed in the hub under the control of *upd* or *hh* promoter, where it mediates the recombination of the two FRT sites to excise the STOP cassette, leading to constitutive expression of Stinger (GFP) under the control of the *ubiquitin* (*Ubi*) promoter in hub cell and its progeny. Somatic cyst cell driver *c587Gal4* is used to manipulate gene expression in CySCs. (B)–(E) Lineage tracing of hub cells in control and *c587* ^*ts*^ *>esg* ^*RNAi*^ testes at 29°C for indicated time points. Some hub cells (white dotted cycles) are marked by GFP in control and these GFP^+^ hub cells are delaminated from the hub and convert into CySCs to produce differentiated cyst cells (white arrowheads) upon *esg* depletion in CySCs. Note that some newly delaminated GFP^+^ cells from the hub still express high levels of FasIII (white arrowheads). FasIII and GFP channels are shown separately. Scale bars: 10 μm. (F) Quantification of the number of GFP^+^ cells outside of the hub per image in testes with indicated genotypes. Mean ± SD is shown. Ordinary one‐way ANOVA test was used. (G) Quantification of the number of hub cells/image in testes with indicated genotypes. Mean ± SD is shown. Ordinary one‐way ANOVA test was used.

We were strongly intrigued by the gradual depletion of hub cells in *c587* ^*ts*^>*esg* ^*RNAi*^ testes and eager to know the destination of hub cells. We speculated that hub cells in *c587* ^*ts*^>*esg* ^*RNAi*^ testes were lost due to either cell death or conversion of their fate into other cell types which were subsequently delaminated from the hub. However, when examined with the apoptosis reporter GC3Ai,^87^ no cell death was observed in the hub of *c587* ^*ts*^ *>esg* ^*RNAi*^ testes, indicating that the loss of hub cells is not caused by cell death (SI Appendix, Figure S5N–P). As our abovementioned data show that *esg* is intrinsically required for CySC maintenance and *esg* depletion in somatic cyst cells results in CySC loss, we hypothesized that hub cells may convert their fate into CySC in response to systemic CySC loss in *c587* ^*ts*^ *>esg* ^*RNAi*^ testes. We tested the hypothesis by tracing the lineage of hub cells in combination with gene knockdown in somatic cyst cells by c*587* ^*ts*^ (G‐TRACE, Figure 4A). We developed an modified version of G‐TRACE technique to specifically label the lineage of hub cells, we generated FLPase that is specifically expressed in the hub (by either *upd‐flp* or *hh‐flp*) and utilised the *Ubi>STOP>Stinger* cassette to specifically label hub cells, the STOP cassette will be removed in the presence of FLPase and constitutive GFP (Stinger) will be expressed in the labelled cells and their progeny (Figure 4A). GFP‐labelled cells could only be detected in the hub of control testis by this G‐TRACE technique (Figure 4B and SI Appendix, Figures S8A and S9A). Interestingly, some GFP^+^ cells were detected at regions away from the hub after depleting *esg* within CySCs for only 2 days (Figure 4C). These GFP^+^ cells away from the hub were somatic cyst cells as they were negative of germline cell marker Vasa and encapsulated germline cells (Figure 4C).^88^ It was worth to note that some of these GFP^+^ cells away from the hub still expressed high levels of FasIII (the widely used hub marker) (Figure 4C). At 5 days of *esg* depletion in CySCs, increased number of GFP^+^ cells appeared at regions further away from the hub, accompanied by obvious loss of hub cells (Figure 4B–D, F, G). Till 8 days of *esg* depletion in CySCs, nearly all GFP^+^ cells were scattered in the testes, with undiscernible hubs (Figure 4E). GFP^+^ cells were located further away from the apex of the testis, forming differentiating cysts (Figure 4E). Quantification data show that during the tracing period, the number of GFP^+^ cells away from the hub is significantly increased, accompanied by a dramatic reduction in the number of hub cells (Figure 4B–G). Identical phenomena were observed when another hub‐specific FLPase was used (SI Appendix, Figure S8B–F). Furthermore, some of these delaminated GFP^+^ hub‐lineage cells outside the hub expressed high levels of the CySC marker Zfh1, suggesting that they adopt CySC identity (SI Appendix, Figure S9A, B‴). Meanwhile, some of these delaminated cells are Zfh1 negative, indicative of differentiated cyst cells (SI Appendix, Figure S9B). These data show that hub cells begin to delaminate from the hub upon systemic CySC loss and are converted into functional CySCs, but these newly converted CySCs could not be maintained as *esg* is continuously depleted in them, leading to differentiation of these newly converted CySCs. Thus the hub is finally exhausted by repeated rounds of conversion‐differentiation. Taken together, the above data demonstrate that *esg* plays pivotal roles in CySCs to intrinsically maintain CySC fate and may regulate hub cell conversion directly or indirectly, thereby maintaining the homeostasis of the testicular niche.

Herrera et al. showed that CySCs maintained hub quiescence and restricted hub‐to‐stem‐cell trans‐differentiation by secreting Follistatin, an Activin antagonist, to inhibit the Activin signalling in hub cells.^40^ Then, we tested whether Esg in CySC maintains hub quiescence through Activin signalling. We stained for *esg*‐defective testis with antibody phosphorylated SMAD3 (pSMAD3), the homologue of Smox, which is phosphorylated upon activation of the Activin pathway. However, loss of *esg* in CySCs did not affect the levels of pSMAD3 in hub cells, indicating that Esg may not be involved in the Activin pathway to inhibit hub conversion (SI Appendix, Figure S10). Altogether, these data indicate that Esg may function through other signals directly or it may secondarily affect the conversion from hub cells to CySCs.

### 2.5 *socs36E* is a putative downstream target of *esg* and negatively regulated by *esg*

**FIGURE 5:**
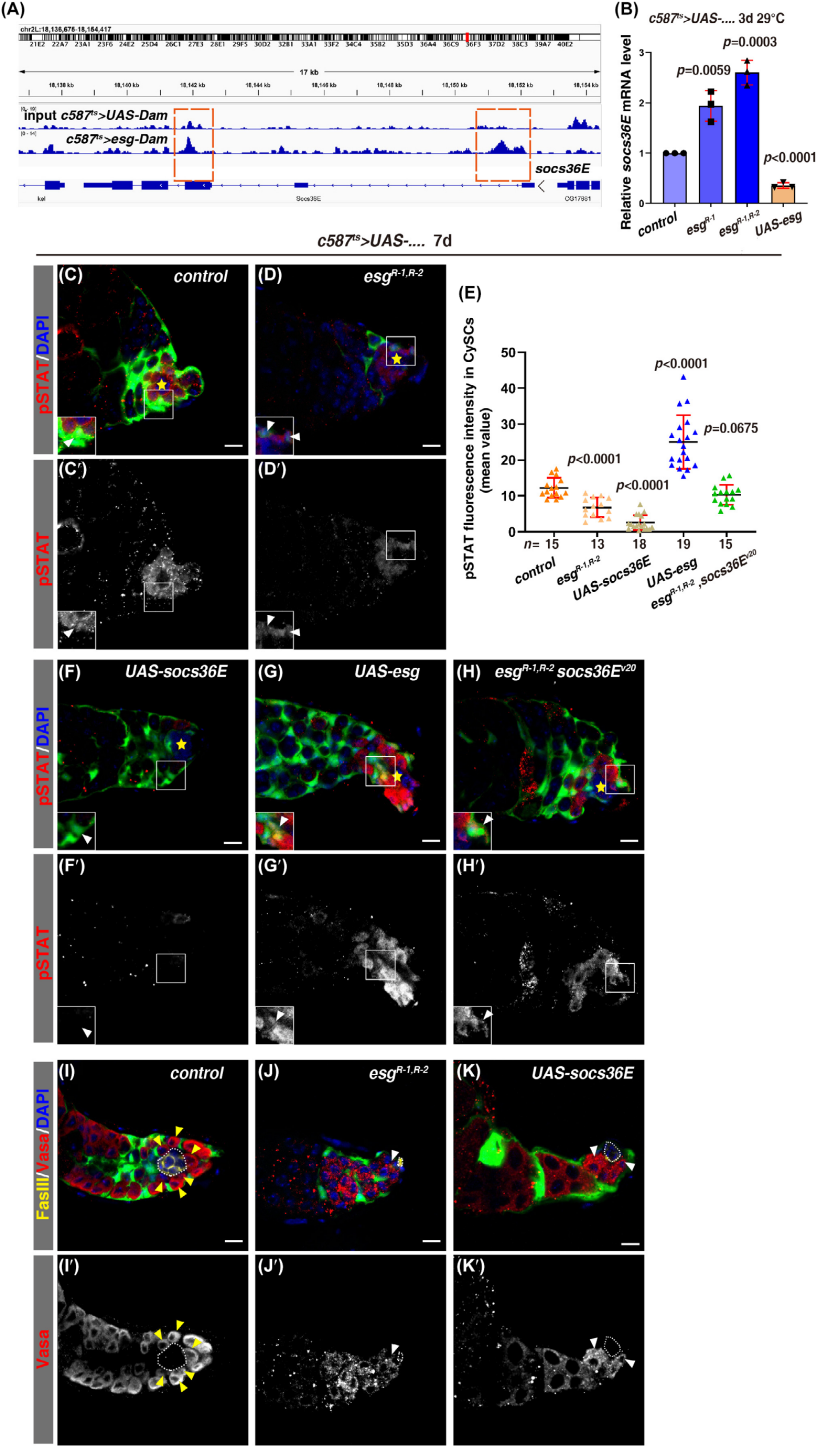
*esg* directly suppresses the expression of *socs36E*. (A) Dam‐ID analysis for Esg‐Dam and control reveals binding peaks of Esg at the *socs36E* region (orange dashed boxes). (B) qRT–PCR quantification of *socs36E* mRNA levels in testes with indicated genotypes at 29°C for 3 days. Mean ± SD is shown. Two‐tailed Student's *t*‐test was used. *n* = 3. (C), (D) pSTAT (red) in control and *c587* ^*ts*^ *>esg* ^*RNAi*^ testes at 29°C for 7 days. CySCs (white arrowheads) in boxed region are shown in high magnification. pSTAT channel is shown separately in black and white. (E) Quantification of pSTAT fluorescence intensity in CySCs of testes with indicated genotypes. Mean ± SD is shown. Ordinary one‐way ANOVA test was used. (F)–(H) pSTAT (red) in *c587* ^*ts*^ *>socs36E*, *c587* ^*ts*^ *>esg*, and *c587* ^*ts*^ *>esg* ^*RNAi*^, *socs36E* ^*v20*^ testes at 29°C for 7 days. CySCs (white arrowheads) in boxed region are shown in high magnification. pSTAT channel is shown separately in black and white. (I)–(K) Immunostaining of Vasa (red) and FasIII (yellow) in control, *c587* ^*ts*^ *>esg* ^*RNAi*^, and *c587* ^*ts*^ *>socs36E* testes from at 29°C for 7 days. Overexpression of *socs36E* mirrors that of *esg* depletion. In all confocal images, GFP (*c587>GFP*) labels somatic cyst cells, the hubs are indicated by yellow asterisks or dotted cycles, the germline cells are stained by Vasa, and DAPI stains the nucleus in blue. Scale bars: 10 μm.

To determine how Esg controls CySC maintenance and niche homeostasis, putative targets of Esg in somatic cyst cells were systemically identified through Dam‐ID technology as Esg is a Snail family transcription factor. An Esg‐Dam fusion protein was expressed in somatic cyst cells by *c587* ^*ts*^ and genome‐wide Esg‐binding peaks were determined. Interestingly, we found that Esg binds on the gene loci of *ImpL2* and *InR* in our Dam‐ID result, as previously reported (SI Appendix, Figure S12),^80^ proving the effectiveness of our Dam‐ID results. Unexpectedly, we found that Esg bound to sites within *socs36E* transcription units, indicating that *socs36E* is a putative downstream target of Esg (Figure 5A). In order to confirm whether Esg is sufficient to regulate the expression of *socs36E*, qRT‐PCR, and immunostaining experiments were further performed. The qRT‐PCR results showed that the levels of *socs36E* transcripts were significantly increased upon *esg* depletion (Figure 5B). By contrast, the mRNA levels of *socs36E* were dramatically decreased upon *esg* overexpression (Figure 5B). Consistently, we found that the levels of the mean fluorescence intensity of SOCS36E protein in CySCs and cyst cells were significantly increased in *c587* ^*ts*^>*esg* ^*RNAi*^ testes compared to those in control (SI Appendix, Figure S11). These results showed that *socs36E* is a putative downstream target of Esg and negatively regulated by Esg under physiological conditions. As *socs36E* is one of the best characterised JAK/STAT target genes and encodes a negative regulator of JAK/STAT signalling,^36^, ^56^, ^60^, ^62^, ^89^ we then examined whether Esg controls the maintenance of CySC fate and niche homeostasis through the SOCS36E‐JAK/STAT signalling axis. Compared with those in control testes, the levels of phosphorylated STAT (pSTAT, indicator of JAK/STAT signalling activation) were significantly decreased in CySCs of *c587* ^*ts*^>*esg* ^*RNAi*^, indicating that Esg may function through the SOCS36E‐JAK/STAT signalling axis to maintain CySC and niche homeostasis (Figure 5C–E).^51^, ^90^ Supporting this notion, ectopic expression of *socs36E* in CySCs also dramatically decreased the levels of pSTAT (Figure 5A–F). The mRNA levels of *socs36E* in the testes of *c587* ^*ts*^>*socs36E* and *c587* ^*ts*^>*socs36E* ^*RNAi*^ were further confirmed by qRT‐PCR (SI Appendix, Figure S13A). On the contrary, the levels of pSTAT were significantly increased in CySCs ectopically expressing *esg* (Figure 5E, G). To exclude the possibility that the activity of Gal4 may be diluted in the presence of multiple transgenes, we simultaneously expressed the same number and type of transgenes in *esg* knockdown background as control. The results showed that simultaneous expression of the same number and type of control UAS transgenes did not dilute the activity of Gal4 and did not rescue the *esg*‐defective phenotype (SI Appendix, Figure S13B–E). Consistently, further depletion of *socs36E* by an effective RNAi construct against *socs36E* totally restored the levels of pSTAT in CySCs of *c587* ^*ts*^>*esg* ^*RNAi*^ testes (Figure 5C–E, H). These data show that Esg suppresses *socs36E*, thereby promoting JAK/STAT signalling to maintain CySC and niche homeostasis under physiological conditions. In support of this notion, simultaneous expression of STAT totally rescued the defects observed in *c587* ^*ts*^ *>esg* ^*RNAi*^ testes (SI Appendix, Figure S14).

### 2.6 Esg maintains CySC fate and niche homeostasis through SOCS36E

**FIGURE 6:**
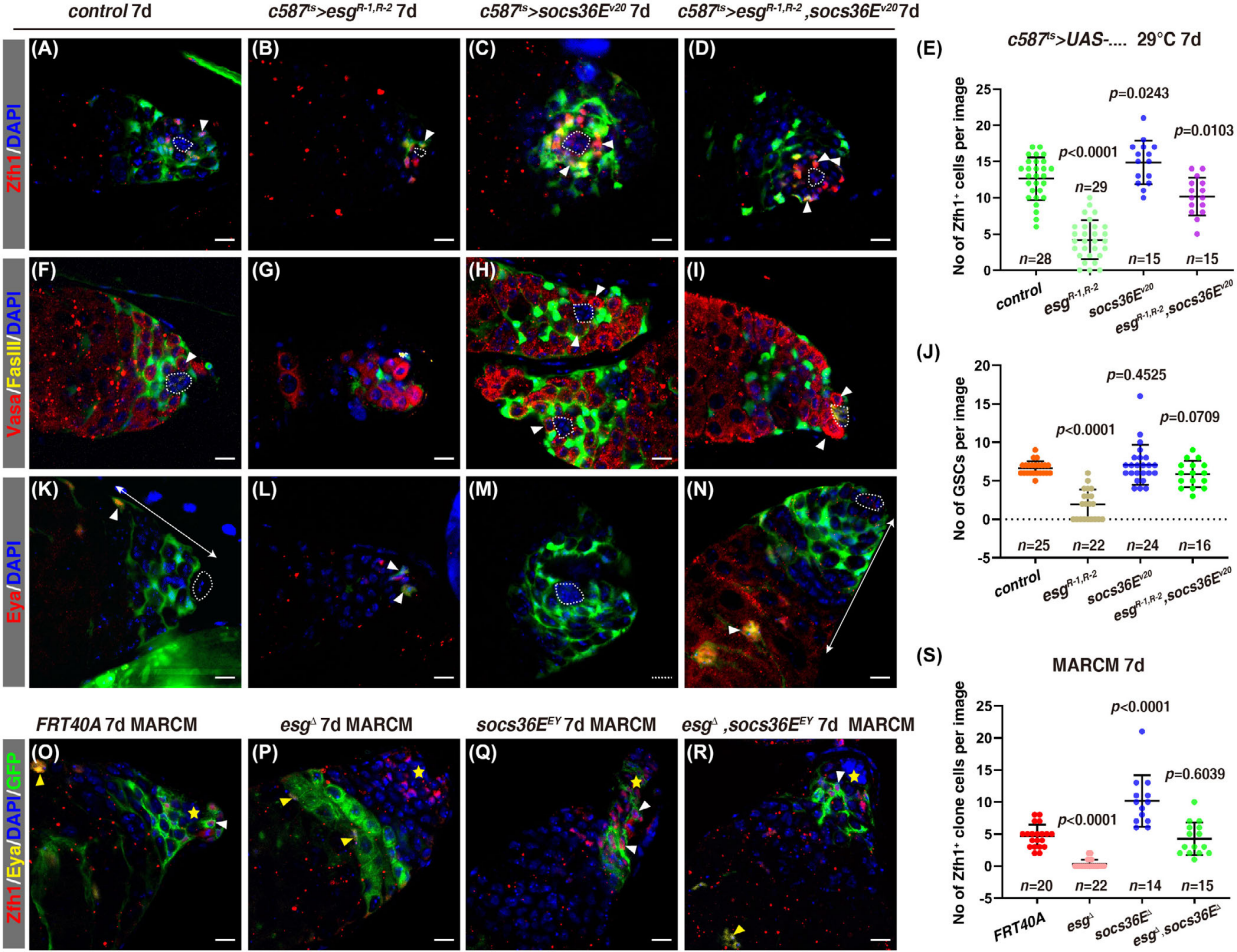
The defects observed in *c587* ^*ts*^ *>esg* ^*RNAi*^ testes are totally restored by loss of *socs36E*. (A)–(D) Zfh1 staining (red, white arrowheads) in testes with indicated genotypes at 29°C for 7 days. (E) Quantification of the number of Zfh1^+^ cells/image in testes with indicated genotypes. Mean ± SD is shown. Ordinary one‐way ANOVA test was used. (F)–(I) Immunostaining of Vasa (red) and FasIII (yellow) in testes with indicated genotypes at 29°C for 7 days. GSC is indicated by white arrowhead. (J) Quantification of the number of GSCs/image in testes with indicated genotypes. Mean ± SD is shown. Ordinary one‐way ANOVA test was used. (K)–(N) Eya staining (red, white arrowheads) in testes with indicated genotypes at 29°C for 7 days. The white lines with double arrowheads indicate the distance between Eya^+^ cyst cells and the hub. (O)–(R) Zfh1 (red, white arrowheads) and Eya (yellow, yellow arrowheads) staining in CySC MARCM clones (green) with indicated genotypes 7 days ACI. (S) Quantification of the number of Zfh1^+^ cells/clone in testes with indicated genotypes. Mean ± SD is shown. Ordinary one‐way ANOVA test was used. In all confocal images, yellow asterisks or white dotted cycles mark the hub, and DAPI stains the nucleus in blue. Scale bars: 10 μm.

Consistent with the notion that Esg suppresses *socs36E* to maintain CySC and niche homeostasis, ectopic *socs36E* expression in CySCs phenocopied the defects observed in *c587* ^*ts*^ *>esg* ^*RNAi*^ testes in every aspect (Figure 5I–K). Next, we examined whether the defects observed in *esg*‐*defective* testes were indeed caused by elevated expression of *socs36E*. First, we found that simultaneous depletion of *socs36E* completely rescued the loss of CySCs observed in *c587* ^*ts*^ *>esg* ^*RNAi*^ testes (Figure 6A–E). Second, the non‐cell autonomous loss of GSCs observed in *c587* ^*ts*^ *>esg* ^*RNAi*^ testes was also totally suppressed by co‐depletion of *socs36E* (Figure 6F–J). Third, consistent with the restoration of CySCs, precocious CySC differentiation observed in *c587* ^*ts*^ *>esg* ^*RNAi*^ testes was also completely rescued by simultaneous *socs36E* depletion (Figure 6K–N). Fourth, the disappeared hub in *c587* ^*ts*^ *>esg* ^*RNAi*^ testis was completely restored by co‐depletion of *socs36E* (Figure 6A–D, F–I, K–N). Fifth, the loss of CySCs observed in *esg* ^*Δ*^ CySC MARCM clones was completely rescued in CySC MARCM clones of *esg* ^*Δ*^, *socs36E* ^*EY*^ double mutant (Figure 6O–S and SI Appendix, Figure S15). Altogether, these data demonstrate that JAK/STAT downstream target Esg maintains CySC fate and niche homeostasis by suppressing SOCS36E.

## 3 DISCUSSION

**FIGURE 7:**
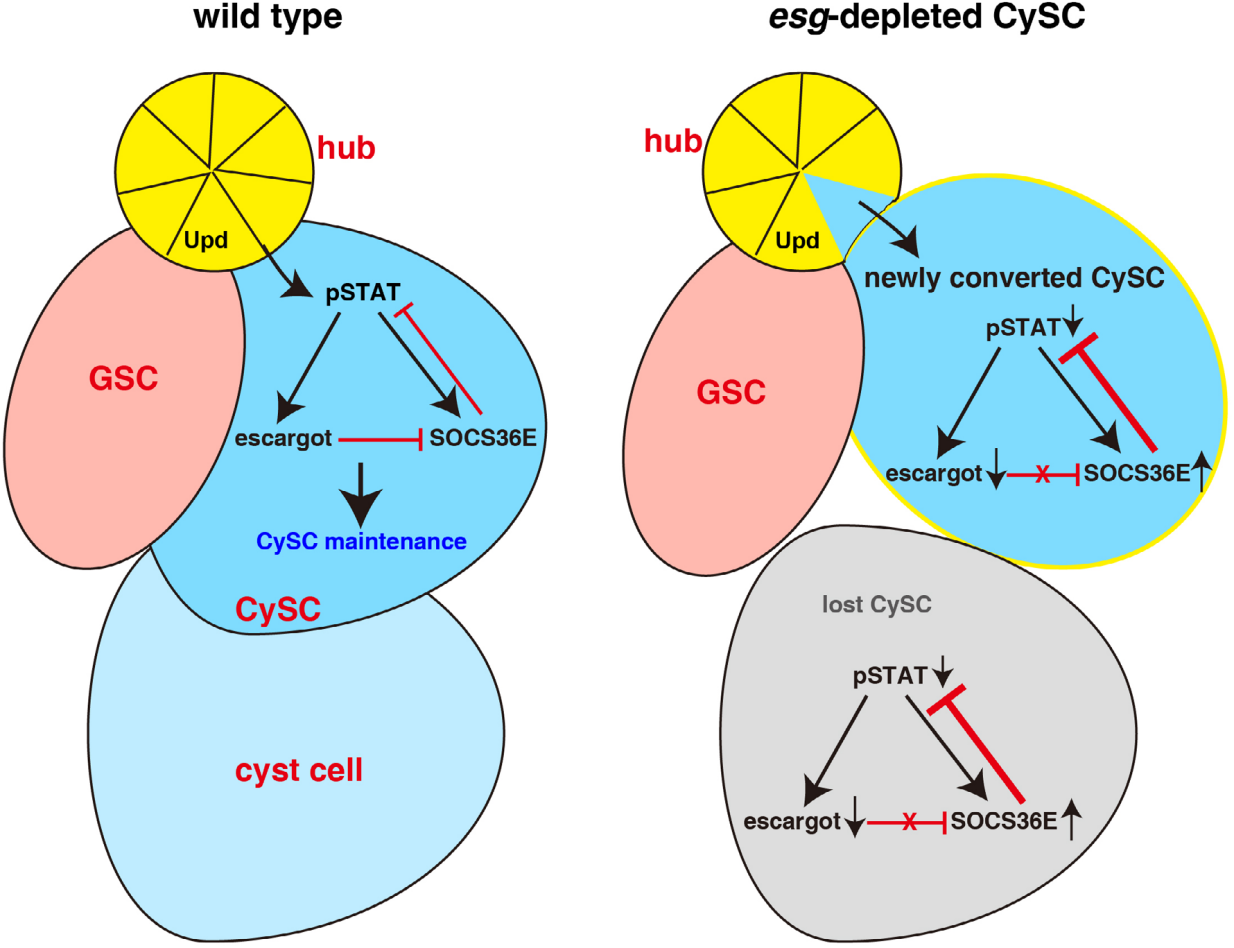
Model of how *esg* maintains CySC fate and prevents hub conversion into CySCs. In wild‐type testis, Upd is produced in the hub (yellow) and activates STAT signalling (by pSTAT) in CySCs (dark blue) to regulate the expression of its downstream targets such as *esg* and *socs36E* to maintain the fate of CySCs and GSCs (pink). Meanwhile, Esg suppresses the expression of SOCS36E, the downstream target and negative regulator of JAK/STAT signalling. Therefore, *socs36E* is regulated by both JAK/STAT signalling and Esg to maintain the homeostasis of the testicular niche. Loss of *esg* in CySCs de‐represses the expression of *socs36E* which in turn suppresses JAK/STAT signalling, leading to systemic CySC loss. Upon the hub senses the systemic loss of CySCs, some hub cells are delaminated from the hub and converted into functional CySCs to replace the lost CySCs. However, these newly converted CySCs could not be maintained as *esg* is continuously depleted in them which induces repeated rounds of hub cell‐CySC conversion, leading to hub exhaustion. A regulatory loop between JAK/STAT signalling, its downstream targets *esg*, and *socs36E* (JAK/STAT‐Esg‐SOCS36E‐JAK/STAT) maintains the proper stem cell niche in adult testis. Thus, our data reveal a regulatory loop in maintaining the homeostasis of adult testicular niche.

The balanced proliferation and differentiation of adult stem cells must be tightly controlled to maintain tissue homeostasis and prevent tumorigenesis and/or degeneration/aging. Stem cell niche provides the required physical environment and extrinsic signals to ensure the self‐renewal (maintenance) and differentiation of residential stem cells, thereby maintaining niche homeostasis. However, how residential stem cells maintain niche homeostasis and whether stromal niche cells could convert their fate into stem cells to replenish lost stem cells upon systemic stem cell loss remain elusive. Here, we identify Esg as a putative JAK/STAT downstream target. Loss of *esg* function in CySCs results in CySC loss and niche eradication. The observations indicate that rather than as passive extrinsic signal recipients from the niche, residential stem cells also actively participate in maintaining niche homeostasis. Esg achieves its roles by suppressing the expression of its downstream target *socs36E*, a well‐known JAK/STAT downstream target and a negative regulator of JAK/STAT signalling.^56^, ^57^, ^60^, ^91^ Thus, our study uncovers a regulatory loop consisting of JAK/STAT‐Esg‐SOCS36E‐JAK/STAT in maintaining the homeostasis of adult testicular niche (Figure 7).

Although it have been showed that JAK/STAT signalling plays important roles in maintaining CySC fate and the homeostasis of the testicular niche, how JAK/STAT signalling fulfils these functions remain largely elusive, albeit a few JAK/STAT downstream targets were identified.^29^, ^51^, ^54^, ^56^, ^57^, ^60^, ^80^, ^89^, ^91^, ^92^, ^93^ Systemic identification of JAK/STAT downstream targets, followed by mechanistic study of these newly identified targets, provides a powerful direction to elucidate these remaining questions. To address these important questions, we systemically identified JAK/STAT downstream targets in adult testis and found a cohort of new JAK/STAT targets, including *p115* and *esg*.^51^ Esg, a member of Snail family transcription factors, has been found in *Drosophila* and mammals to repress differentiation and maintain the stemness of the stem cells by acting as an enhancer or repressor of gene expression.^16^, ^75^, ^76^, ^77^, ^78^, ^79^, ^80^, ^94^ *esg* is previously reported to be essential for maintaining the pool of ISCs and influencing progeny fate decision by repressing *Amun* expression, an inhibitor of Notch signalling in other systems.^78^ Here, we show that *esg* is intrinsically required in CySCs for their maintenance and essential for maintaining niche homeostasis as hub cells (the niche components of CySC) are finally eradicated in testes with *esg‐defective* CySCs (Figures 2, 3, 4). G‐TRACE analyses showed that hub cells were delaminated from the hub and converted into their fate into CySCs to replenish the lost CySCs (Figure 4). To the best of our knowledge, this is the first report that directly monitors the conversion of hub cells into CySCs in vivo upon gradual and systemic CySC loss caused by RNAi knockdown in CySCs. This observation is in clear contrast to what had been observed under physiological conditions in which CySCs are constantly lost and replaced stochastically by neutral competition of neighbouring CySCs.^95^ Thus, niche deploys different manoeuvres in response to stem cell loss under different circumstances: neutral competition of neighbouring CySCs under normal conditions, while niche (the hub) functions as a quiescent stem cell reservoir to replenish depleted CySCs upon systemic stem cell loss as observed in testes with *esg‐defective* CySCs and complete ablation of CySCs.^20^, ^21^ Combined with its roles in intrinsically maintaining the integrity of the hub, previous and our studies show that *esg* plays essential roles in maintaining the homeostasis of adult testicular niche.^16^, ^80^ Meanwhile, although Esg is highly expressed in GSCs, loss of *esg* in GSCs did not cause any obvious defects in GSCs. These observations could be explained by: (1) *esg* alone is not essential for GSC maintenance; (2) in contrast to its essential roles played in CySCs and the hub, *esg* may only play subtle functions in germline cells and spermatogenesis; (3) the defects caused by loss of *esg* in germline cells are somehow counteracted by functional CySCs and the hub.

Here, we find that *socs36E* is a putative downstream target of Esg by Dam‐ID, given the fact that *socs36E* is a well‐known downstream target of JAK/STAT signalling,^56^, ^57^, ^60^, ^89^, ^91^ thus it is natural to propose that *socs36E* integrates multiple upstream signals for stem cell maintenance, differentiation, and niche homeostasis. Consistent with the notions that SOCS36E negatively regulates JAK/STAT signalling through several mechanisms and is de‐repressed in the absence of *esg*,^56^, ^57^, ^58^, ^60^, ^62^, ^91^, ^96^, ^97^ elevated *socs36E* is responsible for the defects caused by loss of *esg* in CySCs (Figures 5 and 6). During this work, *esg* was reported to maintain CySC fate by promoting the expression of *ImpL2*, the fly homologue of the mammal insulin‐like growth factor binding protein 7 (IGFBP7), and suppressing the expression of *InR*, but no indication of hub integrity was showed.^80^ Interestingly, *socs36E* was also found in the Dam‐ID results of that study.^80^ As a member of Snail family transcription factors, Esg regulates the expression of multiple downstream genes.^78^, ^80^ One explanation for the discrepancy between the previous report and our study is that the *esg* ^*RNAi*^ lines used and the time points examined after knockdown are different. Another explanation is that multiple Esg downstream targets are involved in the maintenance of CySCs, while these targets may play different roles in maintaining the homeostasis of niche (hub integrity). The third explanation is that some other *esg* downstream targets function along with ImpL2 and InR to maintain CySC fate as it has been reported that activation of PI3K/Tor signalling alone is not sufficient to drive CySC differentiation.^98^

Considering that both *esg* and *socs36E* are the downstream targets of JAK/STAT signalling and *socs36E* is also the downstream target of Esg, our study shows that Esg positively regulates JAK/STAT signalling by suppressing SOCS36E to maintain CySC fate and niche homeostasis. Thus a feed‐forward loop between JAK/STAT signalling and Esg controls CySC fate and niche homeostasis. Altogether, our data reveal a regulatory cascade showing the interplay among JAK/STAT signalling, its downstream targets *esg*, and *socs36E* in maintaining the proper stem cell niche in adult testis. Interestingly, in our recent study, we uncovered a feed‐forward loop between JAK/STAT signalling downstream target p115 and STAT in GSCs to regulate the maintenance of GSCs,^51^ suggesting that different JAK/STAT downstream targets function in their unique ways to regulate stem cell self‐renewal and differentiation. These findings provide deeper understanding of how JAK/STAT signalling precisely controls the stem cells maintenance. As Esg controls the expression of many other genes, it will be interesting to address whether these genes regulate CySC behaviour in an Esg‐dependent manner. Given the conservation of JAK/STAT signalling, Esg, and SOCS36E, our work provides an insight into the mechanism of how Snail‐family members control stem cell behaviour in other organisms.

## 4 METHODS

### 4.1 Fly lines and husbandry

Flies were maintained on standard media at 25°C. Crosses were raised at 18°C in humidity‐controlled incubators, or as otherwise noted. Information about alleles and transgenes used in this study can be found either in FlyBase or as noted. Please refer to SI Appendix for detailed information.

### 4.2 Immunostainings and fluorescence microscopy

Immunostainings and fluorescent microscopy follow standard procedures as described in SI Appendix.

## AUTHOR CONTRIBUTIONS

R.K., H.Z., and Zhouhua L. designed research; R.K., H.Z., J.L., Y.M., N.L., and Zhouhua L. performed research; R.K., H.Z., L.S., and Zhouhua L. contributed new reagents/analytic tools; R.K., and Zhouhua L. analysed data; R.K., and Zhouhua L. wrote the paper; and Zhouhua L. provided funding.

## CONFLICT OF INTEREST STATEMENT

The authors declare no competing interest.

## Supporting information


**Data S1:** Supporting Information.

## Data Availability

The data that support the findings of this study are openly available in NCBI GEO at https://www.ncbi.nlm.nih.gov/geo/query/acc.cgi?acc=GSE243698, reference number GSE243698.
