# Supplementary material for: A regulatory loop of JAK/STAT signalling and its downstream targets represses cell fate conversion and maintains male germline stem cell niche homeostasis
Source: Cell Prolif. 2024 Jul 10;57(10):e13648. doi: 10.1111/cpr.13648 (PMC11471429; doi:10.1111/cpr.13648)
Supplement: Supplementary file 1 — Data S1: Supporting Information. [file CPR-57-e13648-s001.docx]

**Supplementary Information**

**A regulatory loop of JAK/STAT signaling and its downstream targets represses cell fate conversion and** **maintains male germline stem cell niche homeostasis**

Ruiyan Kong, Hang Zhao, Juan Li, Yankun Ma, Ningfang Li, Lin Shi, Zhouhua Li

**Index of Supplementary Information**

1. Supplementary Figures

2. Supplementary Methods

3. Supplementary References

1. **Supplementary Figures**

**
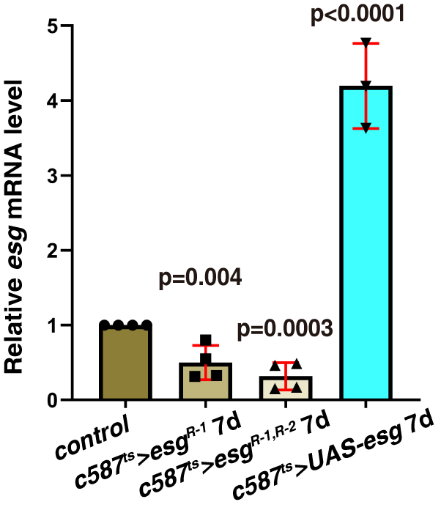
**

**Fig. S1.** **Knockdown efficacy of *esg^RNAi^* lines.**

qRT–PCR quantification of *esg* mRNA levels in testes with indicated genotypes. Mean ± SD is showed. Two tailed Unpaired Student’s *t* test was used. The P value is indicated in the graph. n = 4.


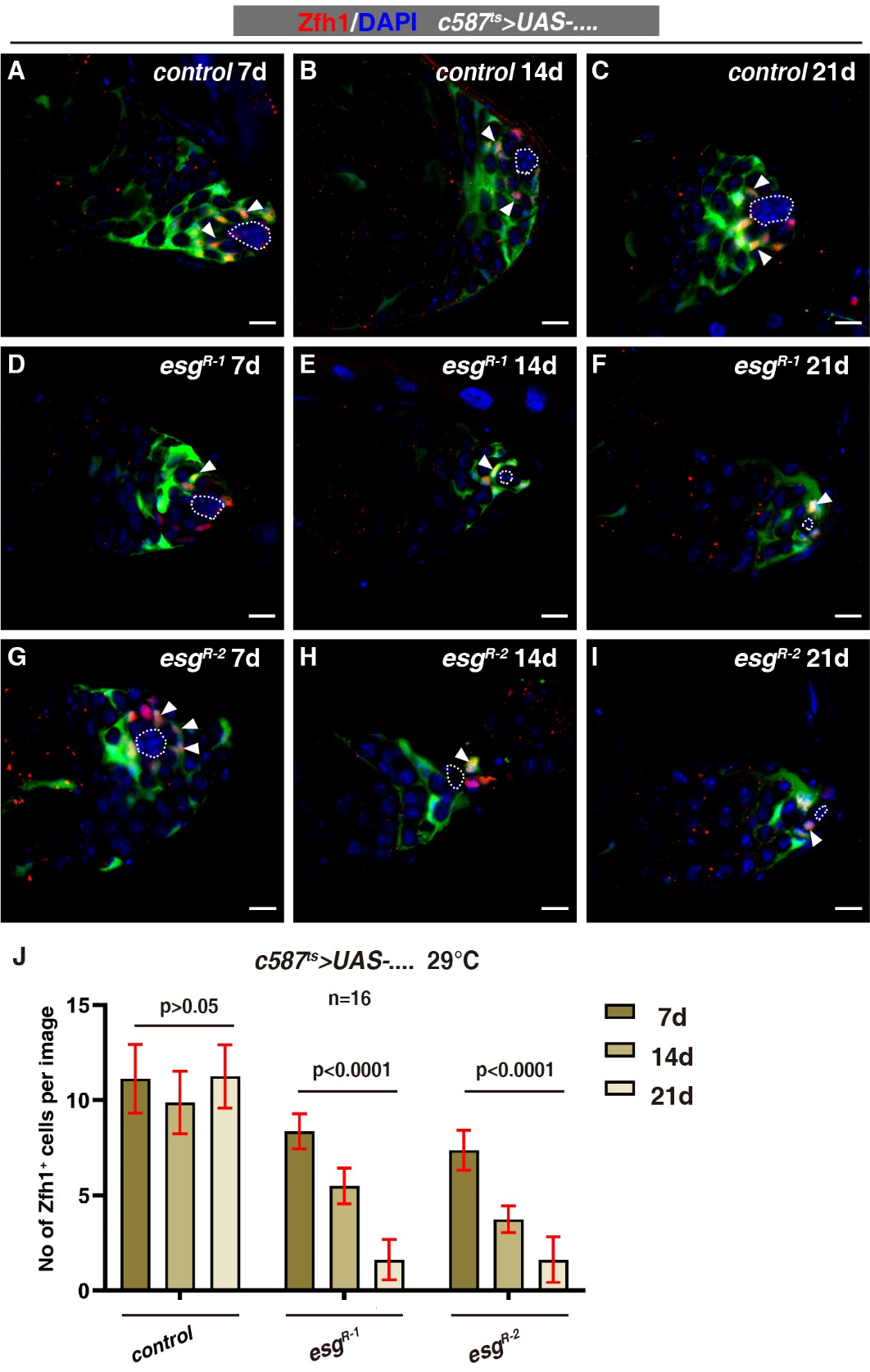


**Fig. S2. *esg* is required for CySC maintenance.**

(*A-I*) Immunostaining of Zfh1 (red, white arrowheads, which is expressed in CySCs and early cyst cells) in control and *c587^ts^ > esg^RNAi^* testes at 29 ºC for 7, 14, and 21 days, respectively. (*J*) Quantification of the number of Zfh1^+^ cells per image in testes with indicated genotypes. Mean ± SD is showed. Multiple Student’s *t* test was used. The P value and n are indicated in the graph (the same as follows).


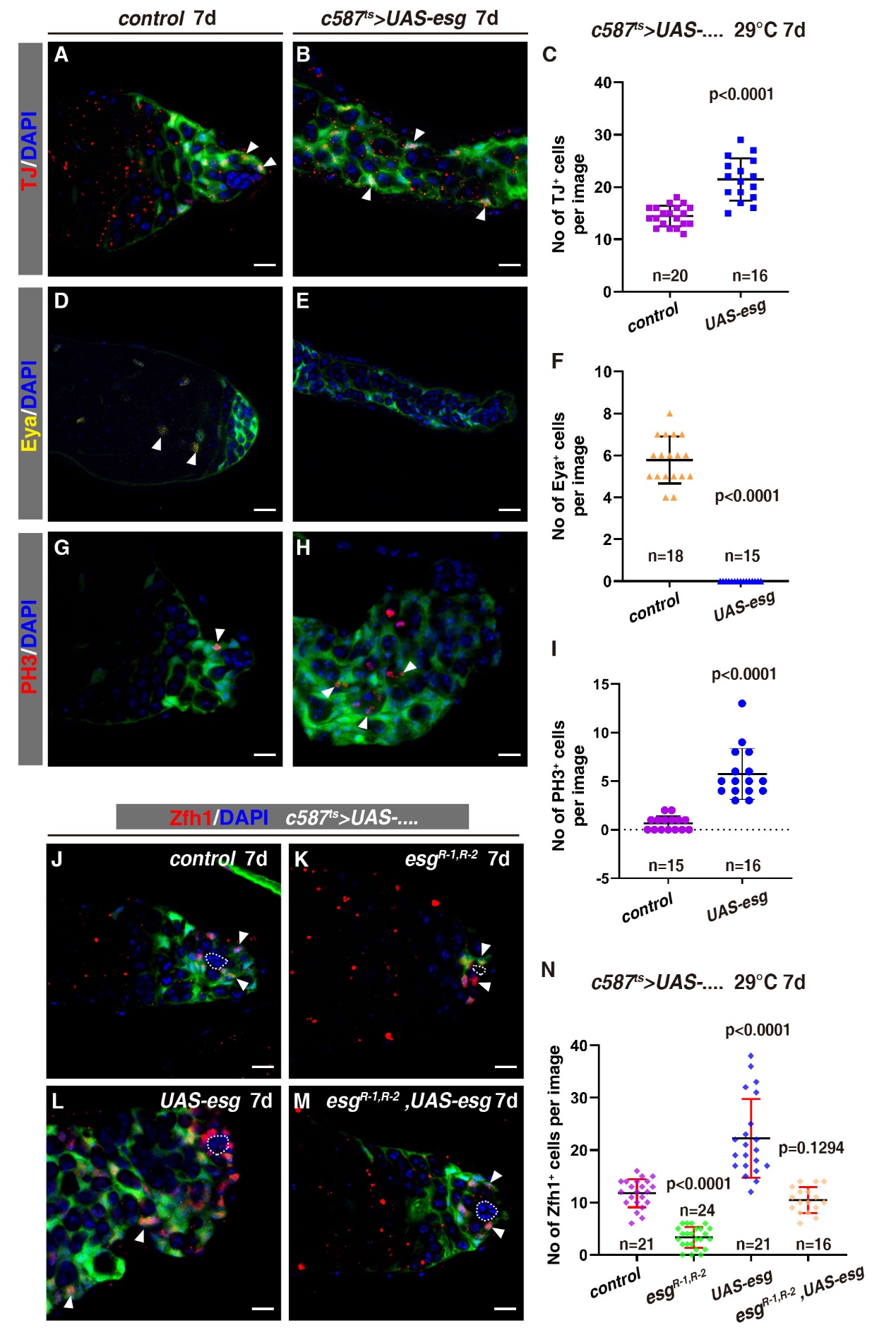


**Fig. S3. *esg* is required for CySC fate maintenance.**

(*A-B*) Immunostaining of TJ (red, white arrowheads) in testes with control and *esg* overexpression genotypes. (*C*) Quantification of the number of TJ^+^ cells per confocal image from testes with indicated genotypes. Mean ± SD is showed. Two-tailed Unpaired Student’s *t* test was used. (*D-E*) Immunostaining of Eya (yellow, white arrowheads) in testes with control and *esg* overexpression genotypes. (*F*) Quantification of the number of Eya^+^ cells per confocal image from testes with indicated genotypes. Mean ± SD is showed. Two-tailed Unpaired Student’s *t* test was used. (*G-H*) Immunostaining of PH3 (red, white arrowheads) in testes with control and *esg* overexpression genotypes (*I*) Quantification of the number of PH3^+^ cells per confocal image from testes with indicated genotypes. Mean ± SD is showed. Two-tailed Unpaired Student’s *t* test was used. (*J-M*) Immunostaining of Zfh1 (red, white arrowheads) in testes with indicated genotypes at 29 ºC for 7 days. (*N*) Quantification of the number of Zfh1^+^ cells per confocal image from testes with indicated genotypes. Mean ± SD is showed. Ordinary one-way ANOVA test was used. In all confocal images, white dotted cycle marks the hub; DAPI (blue) is stained for the nucleus. Scale bars: 10 μm, except for D and E (20 μm).


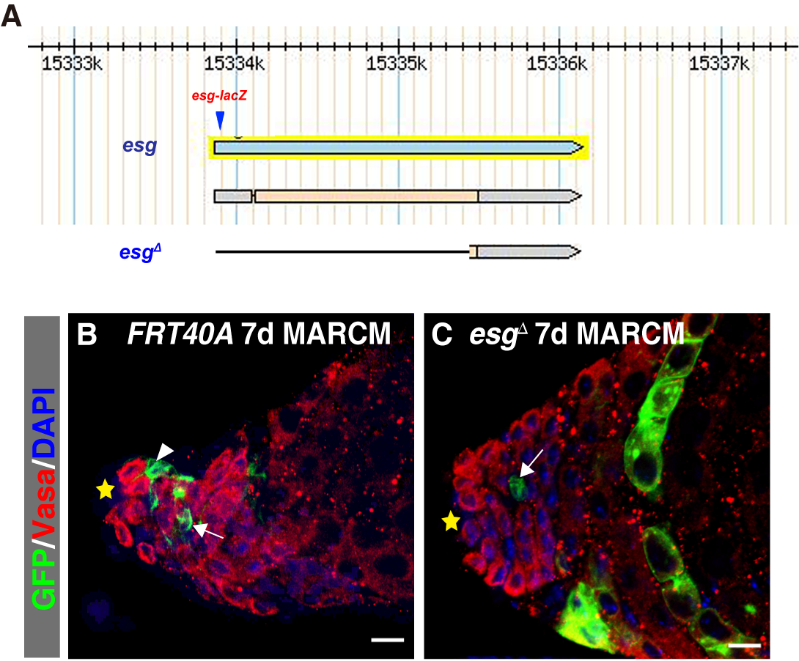


**Fig. S4. Schematic diagram of *esg****^Δ^* **mutant generated and MARCM clonal analysis.**

(*A*) *esg^Δ^* mutant is generated by transposase hopping of the *esg-lacZ* line which removes about 2 kb region from the insertion site to the very 3’ end of *esg* gene (the deleted region is showed with a black line). (*B*-*C*) Immunostaining of Vasa (red) in control and *esg^Δ^* mutant CySC MARCM clones 7 days after clone induction (ACI). The arrowhead indicates CySC and the arrow indicates the differentiated cyst cell in CySC MARCM clones. The yellow asterisk marks the hub; DAPI (blue) is stained for the nucleus. Scale bars: 10 μm.

**
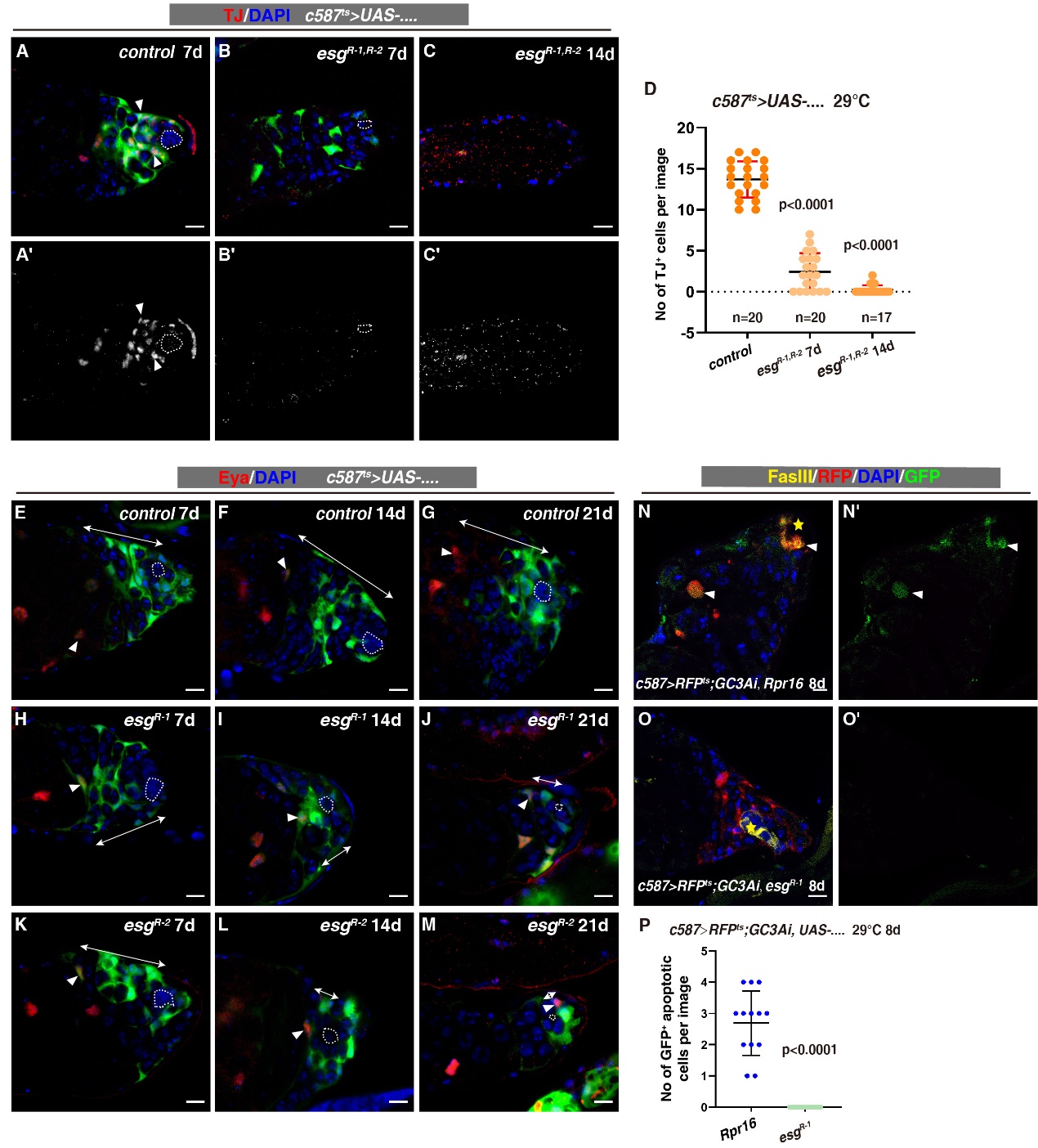
**

**Fig. S5. *esg-*defective CySCs could not be maintained and undergo differentiation.**

(*A-C*) TJ staining (red, white arrowheads, CySCs and early/mid cyst cells) in control and *c587^ts^ > esg^RNAi^* testes at 29 ºC for 7 days and 14 days, respectively. TJ channel is showed separately in black white. (*D*) Quantification of the number of TJ^+^ cells per image in testes with indicated genotypes. Mean ± SD is showed. Ordinary one-way ANOVA test was used. (*E-M*) Eya staining (red, white arrowheads, which is expressed in mature cyst cells) in control and *c587^ts^ > esg^RNAi^* testes at 29 ºC for 7, 14, and 21 days, respectively. The white lines with double arrowheads indicate the distance between Eya^+^ mature cyst cells and the hub. Please note that the hub is diminishing in *c587^ts^ > esg^RNAi^* testes with depletion time increased. (*N-O*) Apoptosis (by GC3Ai in green) could be readily detected in cyst cells expressing *reaper* (*rpr*, involved in the apoptotic signaling pathway) (white arrowheads). However, no cyst cells underwent apoptosis in *587RFP^ts^ > esg^R-1^* testes for 8 days at 29 ºC. GC3Ai (green) channel is showed separately. FasIII (yellow) and asterisk mark the hub; cyst cells are in red by *c587>RFP*. In all confocal images, white dotted cycle or yellow asterisk marks the hub; DAPI (blue) is stained for the nucleus. Scale bars: 10 μm except *N* (5 μm). (*P*) Quantification of the number of GFP^+^ apoptotic cells per confocal image from testes with indicated genotypes. Mean ± SD is showed. Two-tailed Unpaired Student’s *t* test was used.

**
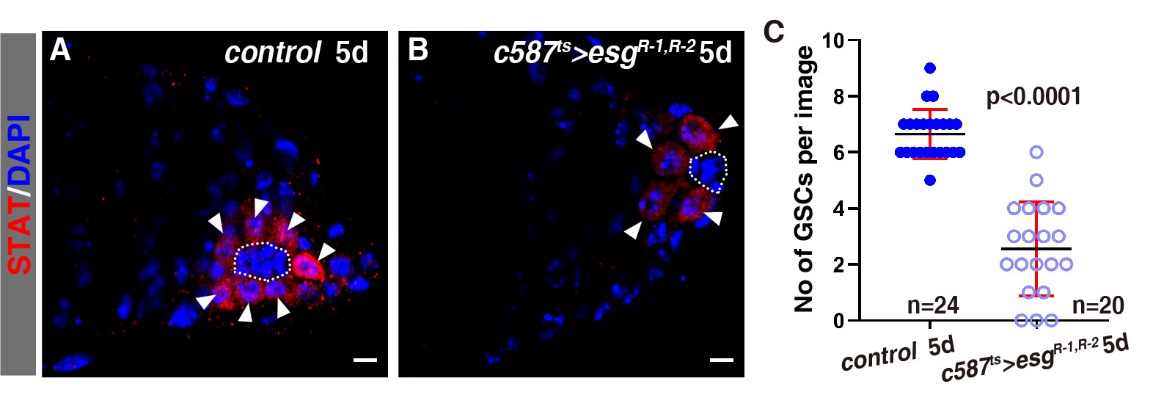
**

**Fig. S6. GSCs cannot be maintained in the absence of *esg* in CySCs.**

(*A-B*) Immunostaining of STAT (red) in control and *c587^ts^ >* *esg^RNAi^* testes at 29 ºC for 5 days. STAT is highly expressed in GSCs (white arrowheads). The hub is indicated by the white dotted cycle; the nucleus is stained by DAPI in blue. Scale bars: 5 μm. (*C*) Quantification of the number of GSCs per confocal image in control and *c587^ts^ >* *esg^RNAi^* testes at 29 ºC for 5 days. Mean ± SD is showed. Two-tailed Unpaired Student’s *t* test was used.

**
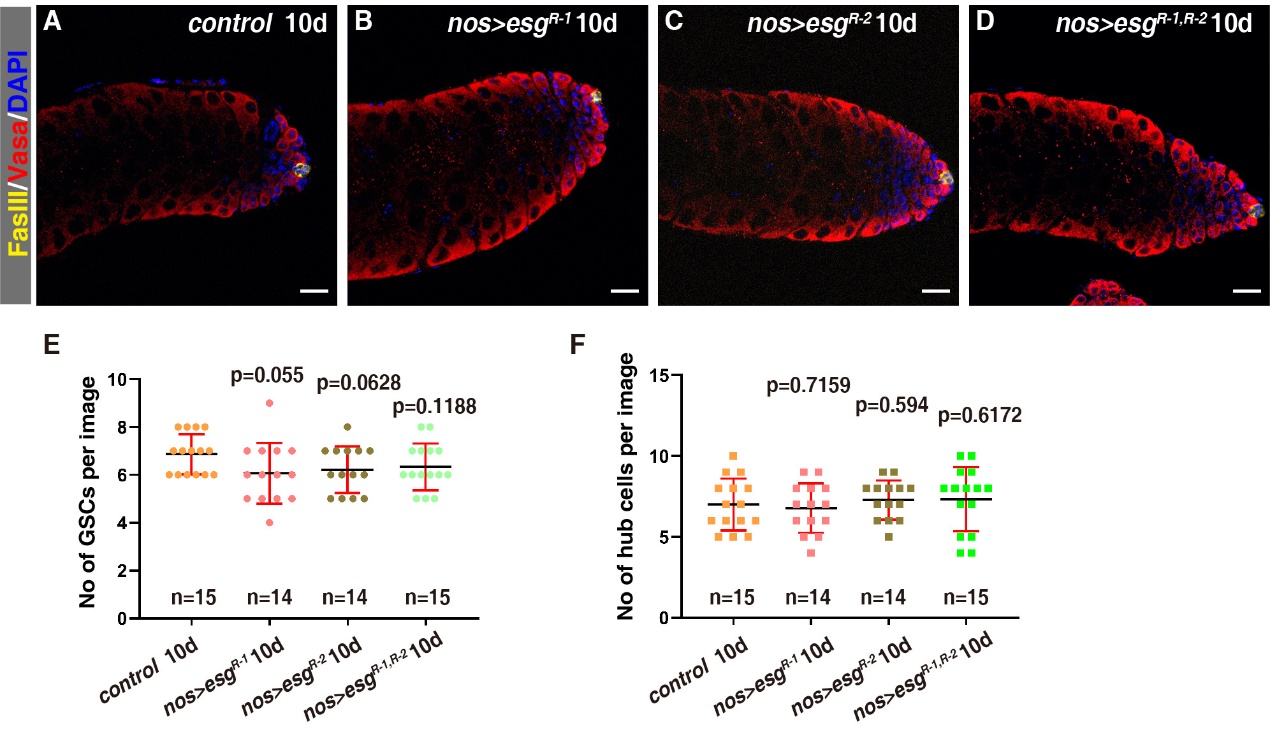
**

**Fig. S7. Knockdown of *esg* in germline cells has no effect on GSC and the hub.**

(*A-D*) Immunostaining of Vasa (red) and FasIII (yellow) in control and *nos >* *esg^RNAi^* testes at 29 ºC for 10 days, respectively. The nucleus is stained by DAPI in blue. Scale bars: 10 μm. (*E*) Quantification of the number of GSCs per confocal image in control and *nos >* *esg^RNAi^* testes. Mean ± SD is showed. Ordinary one-way ANOVA test was used. (*F*) Quantification of the number of hub cells per confocal image in control and *nos >* *esg^RNAi^* testes. Mean ± SD is showed. Ordinary one-way ANOVA test was used.

**
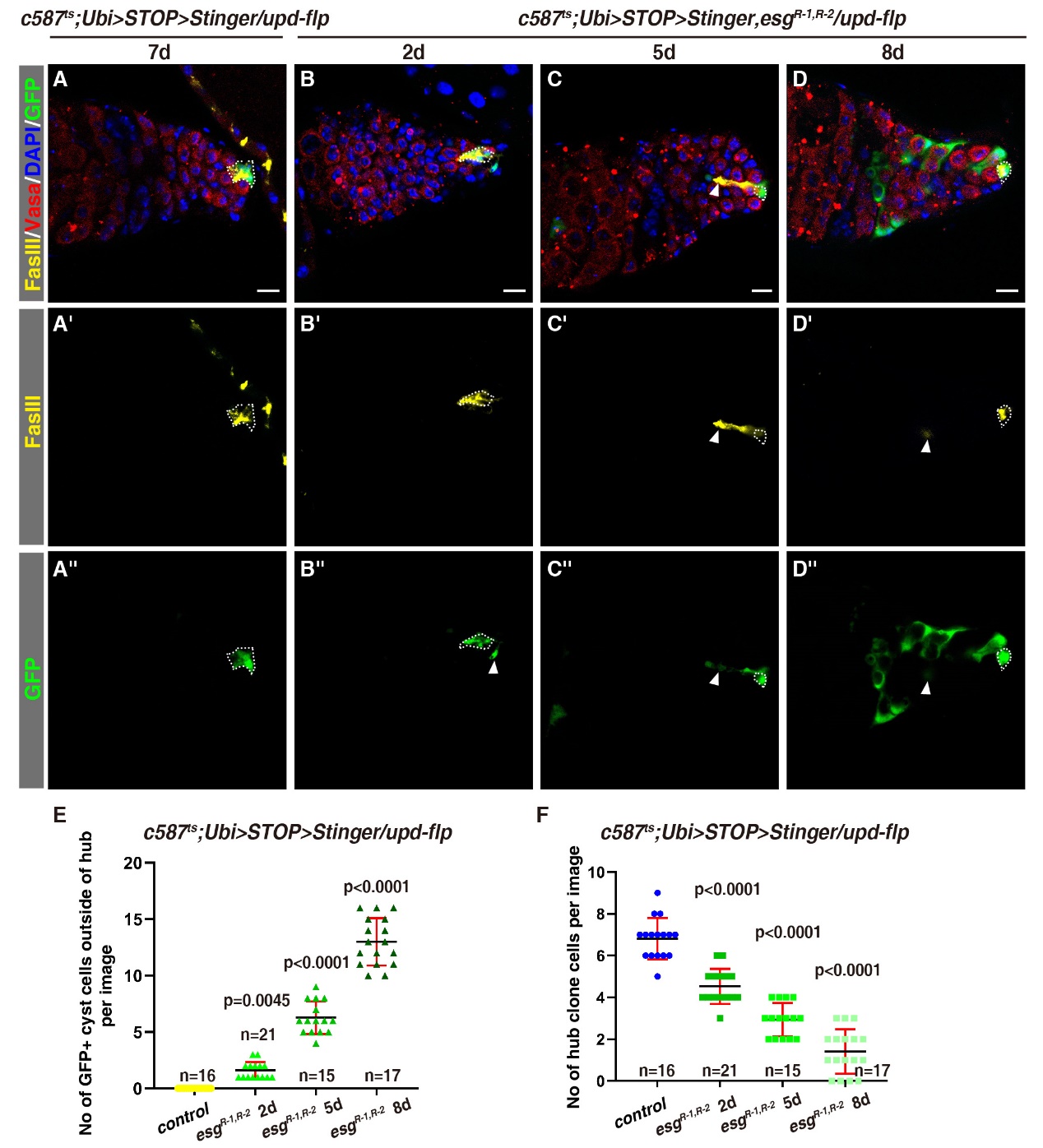
**

**Fig. S8. Loss of *esg* in CySCs promotes the conversion of hub cells into CySCs.**

(*A-D*) Immunostaining of Vasa (red) and FasIII (green) in G-traced control (*A*) and *upd-flp, Ubi>STOP>Stinger, 587^ts^> esg^RNAi^* testes at 29 ºC for 2 days (*B*), 5 days (*C*), and 8 days (*D*), respectively. Compared to control testes in which the GFP^+^ hub cells remained in the hub (white dotted cycles), upon depletion of *esg* in CySCs, GFP^+^ hub cells began to delaminate from the hub and were converted into CySCs (white arrowheads) which can transiently produce differentiated progeny before losing its CySC identity. Please note that some newly delaminated GFP^+^ cells from the hub still express high levels of FasIII (white arrowheads). FasIII and GFP channels are showed separately. The hub is indicated by FasIII in yellow and white dotted cycle, and the nucleus is stained by DAPI in blue. Scale bars: 10 μm. (*E*) Quantification of the number of delaminated GFP^+^ cells from the hub per confocal image in testes with indicated genotypes. Mean ± SD is showed. Ordinary one-way ANOVA test was used. (*F*) Quantification of the number of hub cells per confocal image in testes with indicated genotypes. Mean ± SD is showed. Ordinary one-way ANOVA test was used.

**
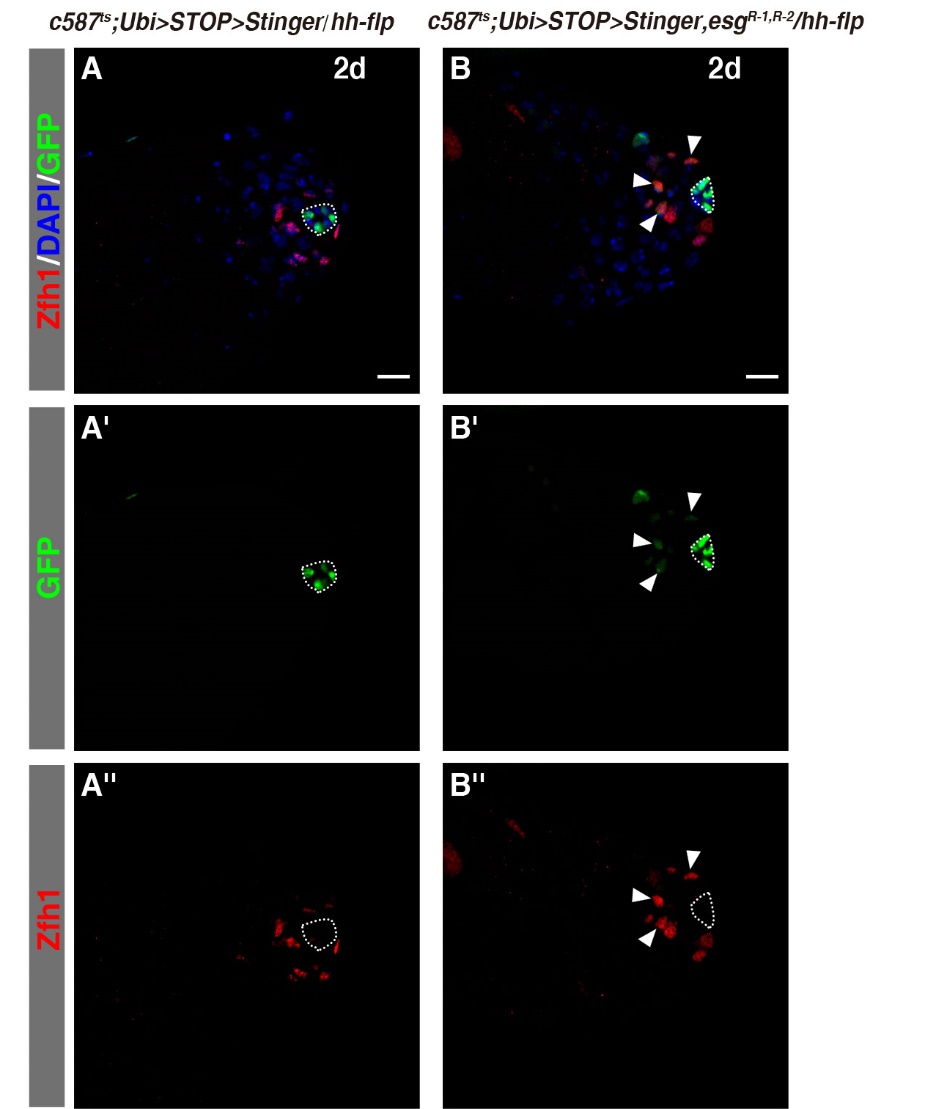
**

**Fig. S9. Loss of *esg* in CySCs promotes the conversion of hub cells into CySCs.**

(*A-B*) Immunostaining of Zfh1 (red) in G-traced control (*A*) and *hh-flp, Ubi>STOP>Stinger, 587^ts^> esg^RNAi^* testes at 29 ºC for 2 day. Compared to control testes in which the GFP^+^ hub cells remained in the hub (white dotted cycles), upon depletion of *esg* in CySCs, GFP^+^ hub cells began to delaminate from the hub and were converted into CySCs (white arrowheads) which can transiently produce differentiated progeny before losing its CySC identity. Zfh1 and GFP channels are showed separately. The hub is indicated by white dotted cycle, and the nucleus is stained by DAPI in blue. Scale bars: 10 μm.

**
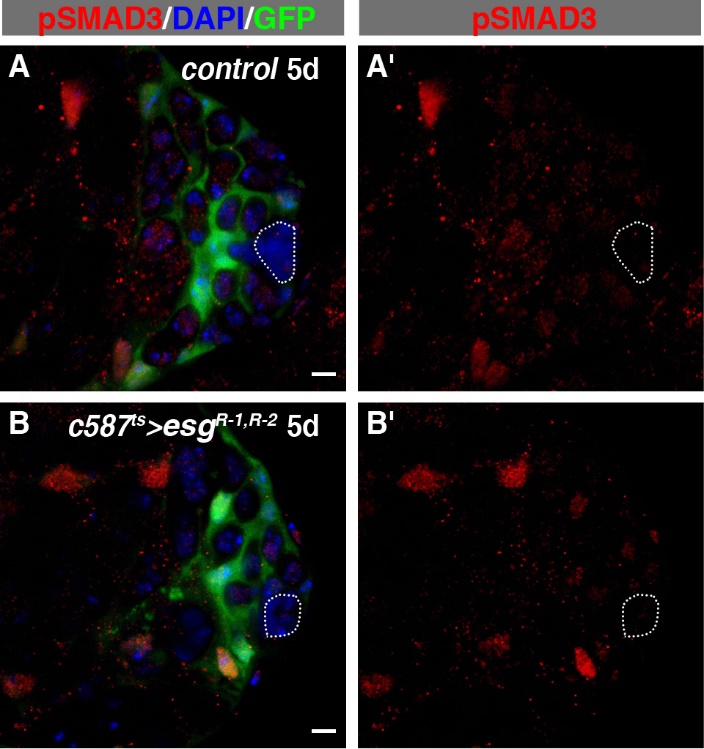
**

**Fig. S10. Esg in CySCs maintains hub quiescence independent of the Activin pathway.**

(*A-B*) Immunostaining of pSMAD3 in testes with indicated genotypes at 29 ºC for 5 days. The hub is indicated by white dotted cycles, and the nucleus is stained by DAPI in blue. Scale bars: 5 μm.

**
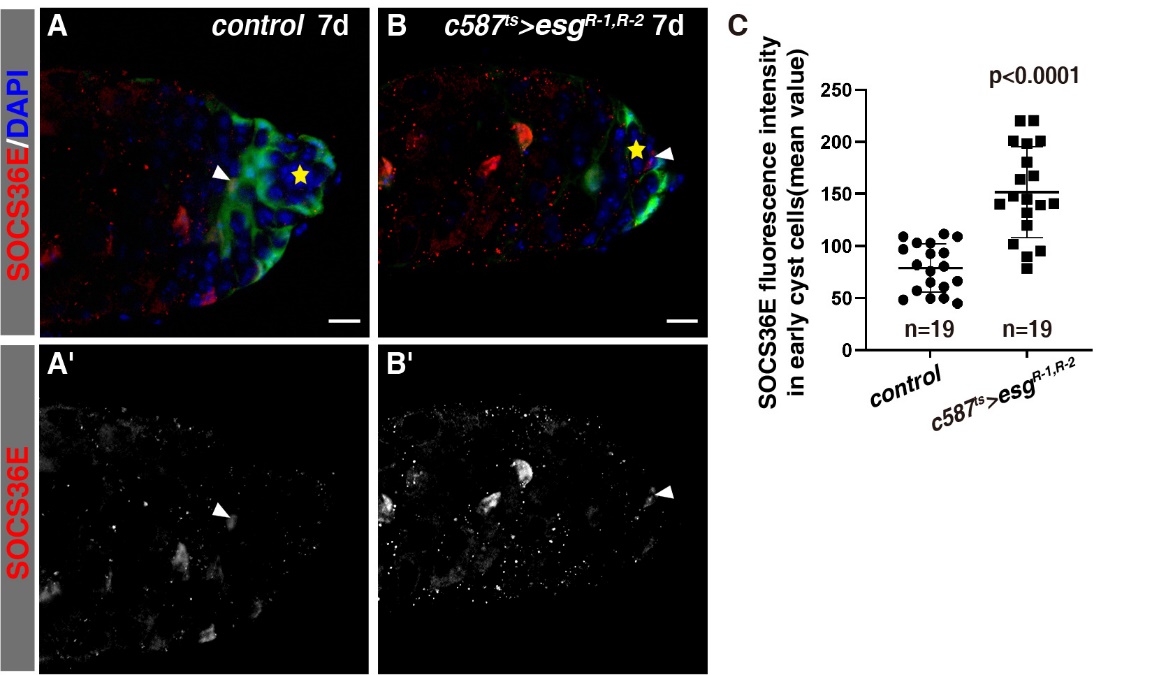
**

**Fig. S11. The levels of socs36E are increased in the absence of *esg* in cyst cells.**

(*A-B*) Immunostaining of SOCS36E in testes with indicated genotypes at 29 ºC for 7 days. SOCS36E channel is showed separately in black white. The hub is indicated by yellow asterisk, and the nucleus is stained by DAPI in blue. Scale bars: 10 μm. (*C*) Quantification of the SOCS36E fluorescence intensity in early cyst cells from testes with indicated genotypes. Mean ± SD is showed. Two-tailed Unpaired Student’s *t* test was used.

**
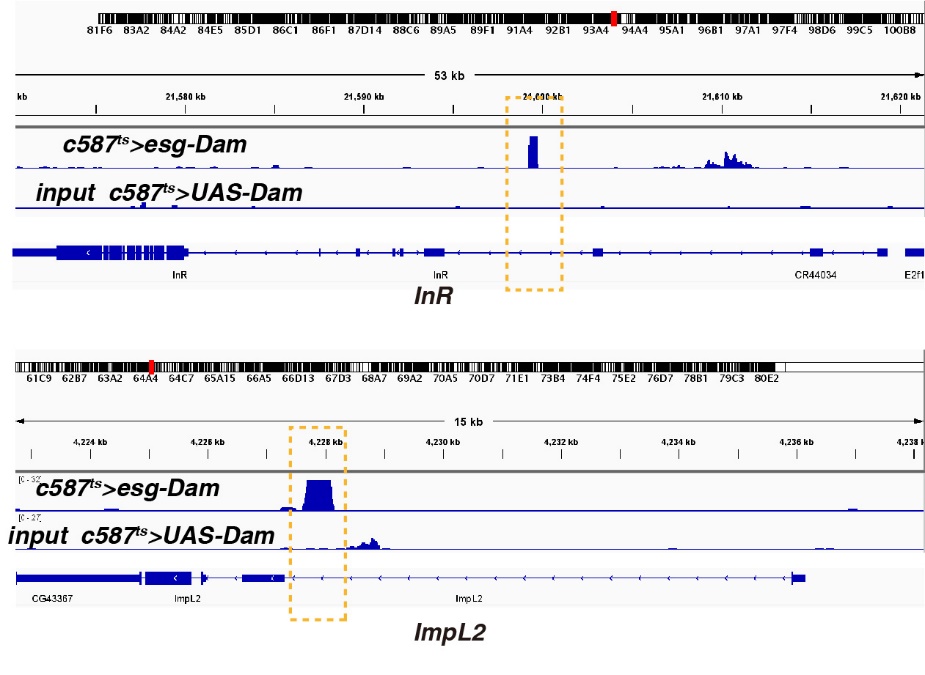
**

**Fig. S12. The other Esg downstream targets identified through our Dam-ID analysis.**

Dam-ID analysis for Esg-Dam and control reveals binding peaks of Esg at the InR and ImpL2 regions (orange dashed box).

**
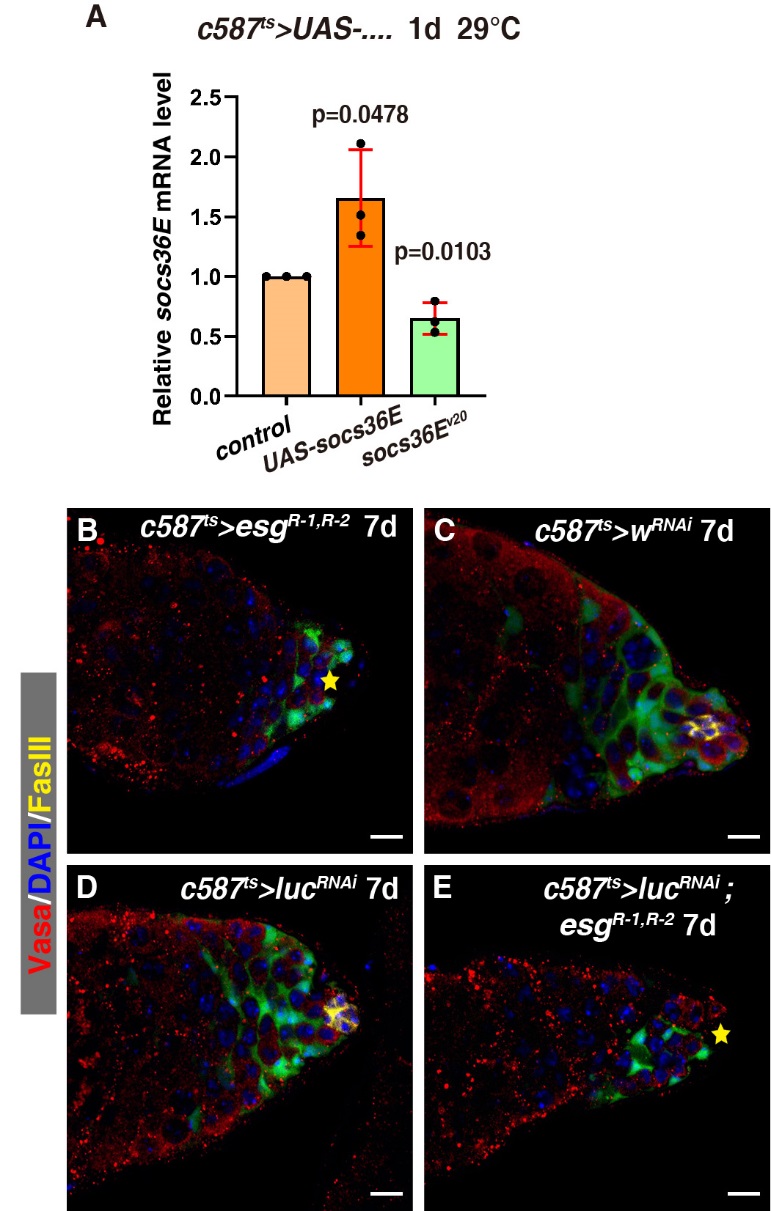
**

**Fig. S13.** **Simultaneous expression of the same number and type of control transgenes does not dilute the activity of Gal4 and does not rescue the *esg*-defective phenotype.**

(*A*) qRT–PCR quantification of *socs36E* mRNA levels in testes with indicated genotypes. Mean ± SD is shown. Two-tailed Unpaired Student’s *t* test was used. n = 3. (*B-E*) Immunostaining of Vasa (red) and FasIII (yellow) in testes with indicated genotypes. Please note that the hub is diminished in *c587^ts^>esg^R-1,R-2^, luc^RNAi^* testis, which is the same to *c587^ts^>esg^R-1,R-2^* testis. *UAS-GFP* is expressed in all testes. The hub is indicated by FasIII and the nucleus is stained by DAPI in blue. Scale bars: 10 μm.

**
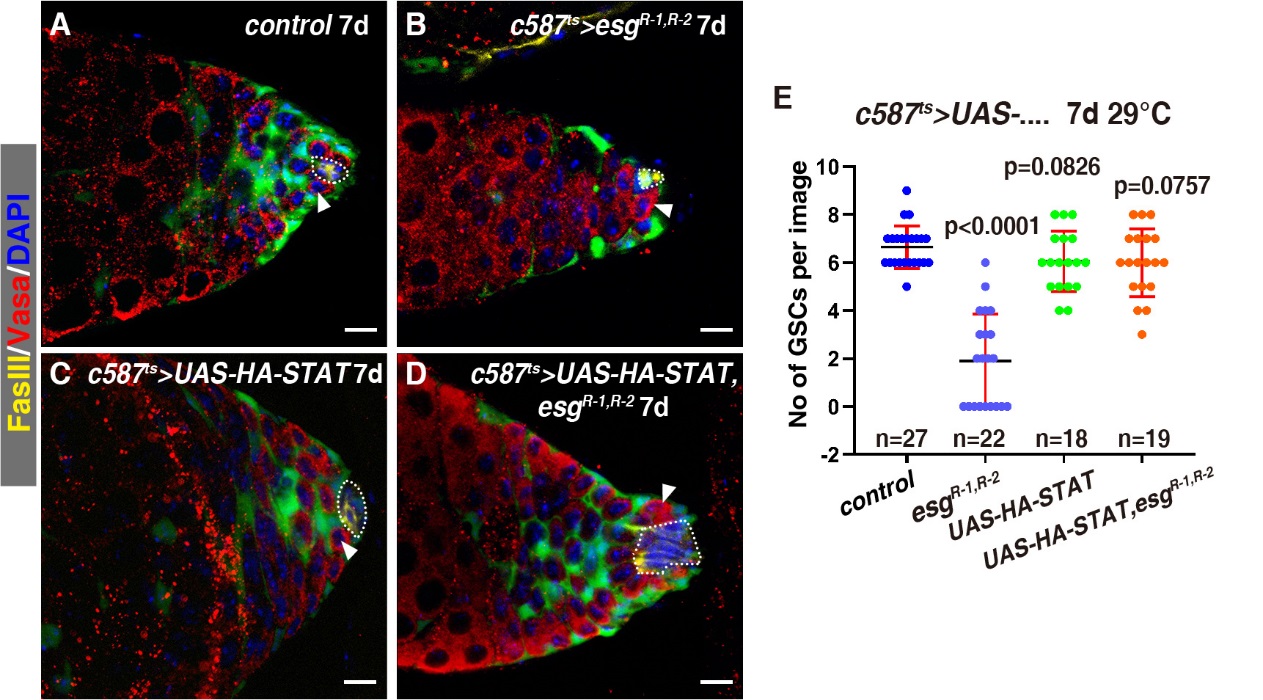
**

**Fig. S14. Ectopic STAT expression completely restores the defects observed in *c587^ts^>esg^RNAi^* testes.**

(*A-D*) Immunostaining of Vasa (red) and FasIII (yellow) in testes with indicated genotypes at 29 ºC for 7 days. GSCs (in red by Vasa, in close contact with the hub) are indicated by white arrowheads. Ectopic STAT expression completely restores the defects observed in *c587^ts^>esg^RNAi^* testes. Somatic cyst cells are labeled by GFP in green (*c587>GFP*), the hub is indicated by FasIII and white dotted cycles, and the nucleus is stained by DAPI in blue. Scale bars: 10 μm. (*E*) Quantification of the number of GSCs per image in testes with indicated genotypes. Mean ± SD is showed. Ordinary one-way ANOVA test was used.

**
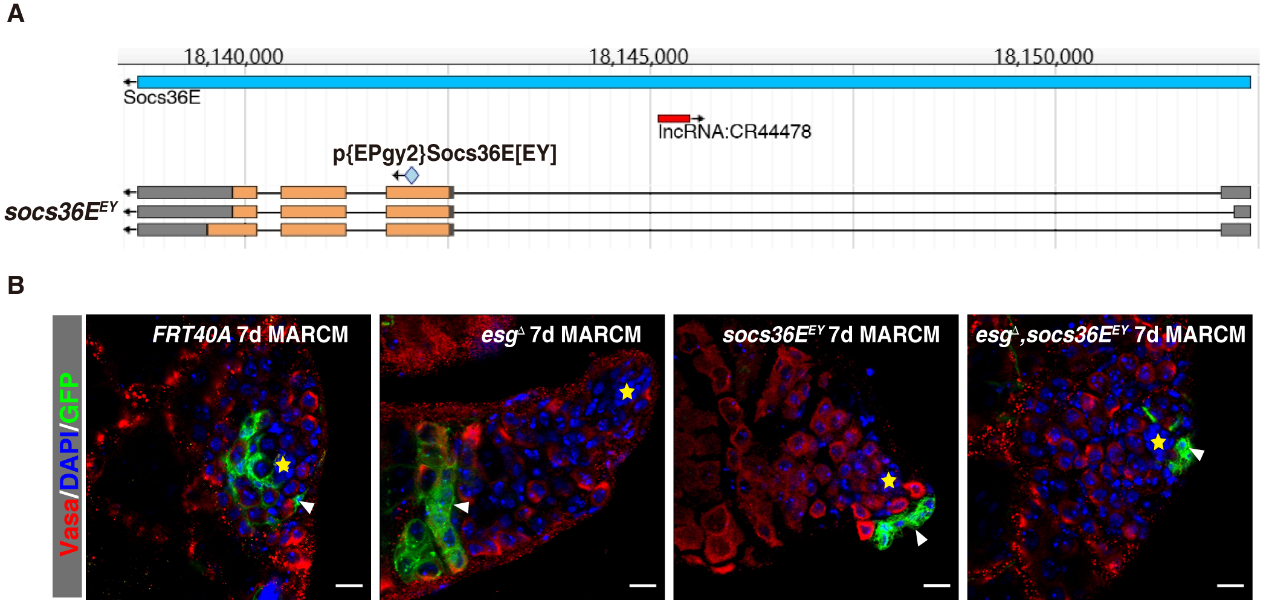
**

**Fig. S15. The defects caused by *esg*-depletion in CySCs can be totally restored by further loss of *socs36E*.**

(*A*) Schematic diagram of *socs36E* mutant, *socs36^EY06665^ (socs36E^EY^* in short). *socs36^EY06665^*, isolated in the gene disruption screen of the Berkeley *Drosophila* Genome Project (FlyBase), carries an EY p-element inserted in the coding exon of *socs36E*. (*B*) Immunostaining of Vasa (red) in CySC MARCM clones (white arrowheads) of control (*FRT40A*), *esg^Δ^* mutant, *socs36E^EY^* mutant, and *esg^Δ^, socs36E^EY^* double mutant 7 days ACI. The hub is indicated by a yellow asterisk and the nucleus is stained by DAPI in blue. Scale bars: 10 μm.

1. **Supplementary Methods**

**Fly Lines and Husbandry.**

Flies were maintained on standard media at 25 ºC. Crosses were raised at 18 °C in humidity-controlled incubators, or as otherwise noted. The desired hatched flies were picked up and transferred to new vials with fresh food every day at 29 ºC and dissected at the time indicated points. Detailed information of the fly stocks used in this study can be found either in FlyBase or as noted: *FRT40A*, *esg-lacZ* (Kyoto, 108851), *nosGal4*, *c587Gal4, UAS-GFP, tubGal80^ts^; esg-lacZ (c587^ts^; esg-lacZ)*, *c587Gal4, UAS-RFP, tubGal80^ts^ (c587RFP^ts^)*, *c587Gal4, tubGal80^ts^ (c587^ts^)*, *UAS-hop^Tum-l^*, *UAS-Dam* (a generous gift from Dr Andrea H. Brand) (1), *esg^R-1^* (NIG. 3758R-1), *esg^R-2^* (THU5422/ HMS0253), *socs36E^v20^* (THU1666/HMS01450), *UAS-esg* (Kyoto, 109127), *P{Δ2-3}* (Kyoto, 106508 and 107128), *UAS-sosc36E* (*Socs36E^G2762^*, BL27006), *P{EPgy2}Socs36E^EY06665^* (BL16744), *UAS-HA-Stat92E* (a generous gift from Dr Erika A. Bach) (2), *UAS-rpr*, *nosGal4; UAS-GC3Ai* (BL84308), *esg-GFP* (a generous gift from L. Cooley), *hsflp, actGal4, UAS-GFP; FRT40A-tubGal80* (for MARCM clonal analysis), *Ubi-p63E(FRT.STOP)Stinger* (*Ubi>STOP>Stinger*, BL32250, BL32251) was used for G-TRACE analysis. *w* (*white*) *^RNAi^* (BL33623, from TRiP at Harvard Medical School) and *luc* (*luciferase*) *^RNAi^* (BL31603) were used as *control.* No significant differences were observed between *c587^ts^>w^RNAi^* and *c587^ts^>luc^RNAi^* when *w^RNAi^* or *luc^RNAi^* was induced in somatic cyst cells. Thereby we mainly used one RNAi line as control.

**RNAi knockdown and overexpression experiments.**

Knockdowns and overexpression were performed by combining the RNAi lines and transgenic stocks respectively with *c587RFP^ts^*, *nosGal4*, *c587^ts^* or *c587^ts^; esg-lacZ* drivers. Crosses were maintained at 18 °C. Flies with desired genotype were collected and transferred to 29 °C. Flies were transferred to new vials with fresh food every day and dissected at time points.

**Generation of *STAT-Dam* and *esg-Dam* transgenic flies.**

To identify JAK/STAT signaling downstream targets, *attB-UAST-STAT-Dam* was constructed. *STAT* ORF was amplified from *UAS-HA-Stat92E* (a generous gift from Dr Erika A. Bach) (2) using primer pair (*STAT-Dam*-F: gatctggccggcgcagatctgcggccgctcA

TGAGCTTGTGGAAGCGCATCGCCAGCC and *STAT-Dam-*R: gtaaggttccttcacaaagat

cctctagaTCAAAAGTTCTCAAAGTTTGTAATCGTATC) and cloned into the NotI and XbaI sites of *attB pUAST-LT3-Dam* (a generous gift from Dr Andrea H. Brand) (1). To identify *esg* downstream targets, *attB-UAST-esg-Dam* was constructed. *esg* ORF was amplified from gDNA using primer pair (*esg-Dam*-F: gatctggccggcgcagatctgcggc

cgctcATGCATACCGTGGAAGACATGTTG and *esg-Dam-*R: gtaaggttccttcacaaagatc

ctctagaTTACGGCTCGGCATAGCCGGCGTAG) and cloned into the NotI and XbaI sites of *attB pUAST-LT3-Dam* (a generous gift from Dr Andrea H. Brand) (1). Transgenic flies were obtained by germline transformation using φΧ31-mediated-site-specific integration with *attP* site at 86F or 36B.

**Generation of *esg* null mutant.**

*esg* null mutant was generated by standard P-element-mediated imprecise excision of *esg-lacZ* in the presence of Δ2-3 transposase. *esg* deletion mutant was identified by PCR using primer pair (*esg*-F: gagcacgatgcacacaggta, *esg*-R: ggcattcggcatagtttcgg). PCR products were sequenced using the corresponding primers to determine the regions deleted. *esg^Δ^* mutant carries a 2 kb deletion from the insertion site to the end of the coding region (*SI Appendix,* Fig. S1).

**Generation of *hh-flp* and *upd-flp* transgenic flies.**

To trace the lineage of hub cells, we selected *hh* and *upd* which are specifically expressed in the hub and generated *hh-flp* and *upd-flp* transgenic lines to specifically express FLP in the hub. *hh-flp* was constructed by cloning the 2.2 kb *hh* promoter and FLP ORF into an *attB* vector. The primers used are: *hh*2.2-5: CCCGGGCGAGAT CT GCGGCCGCGGctcgagccatgggcccgggatggtcttc; *hh*2.2-3: cataatataccaaattgtggCATG ATTTATCTAAGACTCG; *flp/hh*2.2-5: CGAGTCTTAGATAAATCATGccacaatttggt atattatg; *flp*-3: GTAAGGTTCCTTCACAAAGATCCtctagaTTATATGCGTCTATTTA TGTAGGATG. *upd-flp* was constructed by cloning the 2.4 kb *upd* promoter and FLP ORF into an *attB* vector. The primers used are: *upd*2.4-5: CCCGGGCGAGATCTGCG GCCGCGGctcgagCCTGGCCCACTGGCCAGAGATTTC; *upd*2.4-3: cataatataccaaa ttgtggCATGAATGCAACTGCAACCAGAC; *flp/upd*2.4-5: GTCTGGTTGCAGTTG CATTCATGccacaatttggtatattatg; *flp*-3: GTAAGGTTCCTTCACAAAGATCCtctaga TTATATGCGTCTATTTATGTAGGATG. Transgenic flies were obtained by germline transformation using φΧ31-mediated-site-specific integration with *attP* site at 86F or 36B. Both *hh-flp* and *upd-flp* are expressed specifically in the hub.

**G-TRACE analyses of hub lineages**

For G-TRACE analysis of hub cell lineages, we generated transgenic lines specifically expressing FLP in the hub under the control of *hh* or *upd*. *upd-flp, Ubi-p63E(FRT.STOP)Stinger* (*Ubi>STOP>Stinger*)*, c587Gal4, tubGal80^ts^* (*c587^ts^)* and *hh-flp, Ubi>STOP>Stinger, c587^ts^* drivers were developed. To bypass the effect of early Gal4 expression during early developmental stages, all the crosses were raised at 18 ºC. Three days-old males with the desired genotypes were selected to shift to 29 ºC to conduct time-course G-TRACE analyses. The experimental flies were maintained at 29 °C and transferred to new vials with fresh food daily before dissection at indicated time points. Similar results were observed using *hh-flp* and *upd-flp.*

**MARCM analyses.**

Clones were generated using the mosaic analysis with a repressible cell marker (MARCM) system (3). Virgin female flies with genotype of *hsflp, actGal4, UAS-GFP; FRT40A-tubGal80* were crossed with male flies with genotype of *FRT40A*, *FRT40A-esg^Δ^*, *FRT40A-socs36E^EY^* or *FRT40A-esg^Δ^, socs36E^EY^*, respectively. Males with the desired genotypes were selected to conduct time-course clonal analysis after clone induction (ACI). CySC MARCM clones were induced by heat shocking two-day-old males with desired genotypes 1 hour at 37 °C, twice a day for 2 days. The heat shock-treated flies were maintained at 25 °C and transferred to new vials with fresh food daily before dissection at indicated time points.

**Immunostainings and Fluorescence Microscopy.**

Testes were dissected in 1×PBS buffer and fixed in 4% PFA for 20 minutes at room temperature. The samples were then rinsed, washed with 1×PBT (0.1% Triton X-100 in 1×PBS) three times, one time for 5mins and blocked in 3% BSA in 1×PBT for 45 min. The samples were then incubated with primary antibodies at 4 °C overnight. The following primary antibodies were used: rabbit anti-Zfh1 (1:50,000) (4), mouse anti-Eya (10H6, 1:100, developed by Seymour Benzer and Nancy Bonini, DSHB), Rat anti-Tj (1:500, a generous gift from Baeg Gyeong Hun), rabbit anti-Vasa (d-260, 1:200, Cat No: sc-30210, Santa Cruz, USA), rabbit anti-Vasa (1:2,000) (5), mouse anti-FasIII (7G10, 1:100, developed by C. S. Goodman, DSHB), guinea pig anti-Stat92E (1:1,000, a generous gift from Dr Yu Cai), mouse anti-pStat92E (1:2,000, Abmart, China) (6, 7), rabbit anti-β-glactosidase (lacZ, 1:5,000, Cat No: 55978, Cappel, USA), rabbit anti-pSMAD3 (1:500, EPITMICS), rabbit anti-SOCS36E (1:1,000, gift from Steven Hou)(8). The rinsing and washing procedures were conducted and samples were then incubated with the secondary antibodies conjugated with Cy3, 488, or Cy5 (Jackson ImmunoResearch, USA) with a dilution of 1:400 for 2 h at room temperature. DAPI (Sigma, 0.1 μg/mL) was added to the secondary antibodies staining. The samples were mounted in mounting medium (70% glycerol containing 2.5% DABCO). All images were captured using Zeiss LSM 780 laser scanning confocal microscope and processed in Adobe Photoshop and Illustrator.

**Quantitative real-time reverse transcriptase PCR.**

Total RNA was extracted from ~400 testes using TRIzol (Invitrogen, USA) and was cleaned using RNAeasy (QIAGEN, Germany). Extracted total RNA was used to synthesize cDNA through GoTaq® qPCR Master Mix (PROMEGA, USA) according to the manufacturer’s instructions. Oligo (dT)15 primer was used for cDNA library synthesis, which was then used for qPCR. NovoStart® SYBR qPCR SuperMix Plus (Novoprotein, China) was used to perform qPCR following the standard protocol provided by QuantStudio 7 Real-Time PCR System (Applied Biosystems, USA). qPCR was performed in duplicate for each of three independent biological replicates. All results are presented as mean ± SD of the biological replicates. Ribosomal gene *RpL11* was used as normalization control. P-values and data significance was calculated according to two-tailed Student’s *t* test.

**Dam-ID**

Dam-ID was carried out according to a previously described method (9). Testes from *c587Gal4, UAS-GFP, tubGal80^ts^* (*c587^ts^) > STAT-Dam*, *c587^ts^ > esg-Dam* and *c587^ts^ > UAS-Dam* (control) adult fly (about 2,000 flies) were collected after two days expression and immediately preserved on dry ice and kept at -80 °C. Genomic DNA was then isolated and amplified as described in (10). Dam-ID sequence quality was examined by FastQC (version 0.11.9). Dam-ID-seq reads were aligned using Bowtie2 (version 2.5.0) to build version dm6 of the *D. melanogaster* genome. MACS2 (version 2.2.7.1) was used to call peaks from alignment results and to identify regions of enrichment. BigWig files were generated for visualization using the Bioconductor-chipseeker (Version 1.18.0). Raw data from the Dam-ID were submitted to Gene Expression Omnibus (http://ncbi.nlm.nih.gov/geo) with the access number GEO: GSE243698.

**Signal quantiﬁcation**

Image J software was used for signal quantiﬁcation (esg-lacZ, SOCS36E and pSTAT). Two parameters, integrated optical density (IOD) and area, were used in the analysis. IOD value per cell was used. At least 12 different images were analyzed for each sample.

**Data analysis**

The number of Zfh1^+^, TJ^+^ or GSC cells were counted from confocal images of testes with indicated genotypes. Cells expressing high levels of Zfh1 (or TJ) around the hub are regarded as CySCs. The number of GSCs was counted according to Vasa staining, STAT staining, and the position to the hub (GSCs are attached to the hub cells). The number of STAT^+^ and FasIII^+^ cells were counted from images taken at the tips of testes. A marked CySC MARCM clone was calculated by carrying a Zfh1^+^ cell attached to hub cells. The mean fluorescent intensity of esg-lacZ, SOCS36E and pSTAT in CySCs were measured by Image J software. Data processing was analyzed and performed using GraphPad Prism 7.0 (GraphPad Software Inc., USA). P values were determined by two-tailed unpaired Student’s *t* test, multiple Student’s *t* test, Ordinary one-way ANOVA test or mixed two-way ANOVA test. P > 0.05, Not Significant (ns). The number of counted cells/testes (n) and the P values were indicated in the graphs.

**Data Availability**

The data that support the findings of this study are openly available in NCBI GEO at https://www.ncbi.nlm.nih.gov/geo/query/acc.cgi?acc=GSE243698, reference number GSE243698.

**qRT-PCR primers used:**

*esg*-S: CCTACACCGCCGAGTTCTAC

*esg*-A: AGGTAATGATGTGGTGGCGG

*socs36E*-S: TGCTAGTAAAACAATCGGAGGT

*socs36E*-A: AGCTGGCGGTAGGATTTCTC

*RpL11*-S: GGTCCGTTCGTTCGGTATTCGC

*RpL11*-A: GGATCGTACTTGATGCCCAGATCG

1. **Supplementary References**

1. T. D. Southall *et al.*, Cell-Type-Specific Profiling of Gene Expression and Chromatin Binding without Cell Isolation: Assaying RNA Pol II Occupancy in Neural Stem Cells. *Developmental Cell* **26**, 101-112 (2013).

2. L. A. Ekas, G. H. Baeg, M. S. Flaherty, A. Ayala-Camargo, E. A. Bach, JAK/STAT signaling promotes regional specification by negatively regulating wingless expression in Drosophila. *Development* **133**, 4721-4729 (2006).

3. T. Lee, L. Luo, Mosaic analysis with a repressible cell marker (MARCM) for Drosophila neural development. *Trends Neurosci* **24**, 251-254 (2001).

4. R. Xu *et al.*, Self-restrained regulation of stem cell niche activity by niche components in the Drosophila testis. *Dev Biol* **439**, 42-51 (2018).

5. H. Zhao *et al.*, Novel intrinsic factor Yun maintains female germline stem cell fate through Thickveins. *Stem Cell Rep* **17**, 1914-1923 (2022).

6. Y. Zhang, J. You, W. Ren, X. Lin, Drosophila glypicans Dally and Dally-like are essential regulators for JAK/STAT signaling and Unpaired distribution in eye development. *Dev Biol* **375**, 23-32 (2013).

7. R. Kong *et al.*, A feedforward loop between JAK/STAT downstream target p115 and STAT in germline stem cells. *Stem Cell Rep* 10.1016/j.stemcr.2023.08.007 (2023).

8. S. R. Singh *et al.*, Competitiveness for the niche and mutual dependence of the germline and somatic stem cells in the Drosophila testis are regulated by the JAK/STAT signaling. *J Cell Physiol* **223**, 500-510 (2010).

9. J. A. Gutierrez-Triana, J. L. Mateo, D. Ibberson, S. Ryu, J. Wittbrodt, iDamIDseq and iDEAR: an improved method and computational pipeline to profile chromatin-binding proteins. *Development* **143**, 4272-4278 (2016).

10. J. Chen *et al.*, Transient Scute activation via a self-stimulatory loop directs enteroendocrine cell pair specification from self-renewing intestinal stem cells. *Nat Cell Biol* **20**, 152-161 (2018).
